# Effect of chronic intermittent hypoxia on ocular and intraoral mechanical allodynia mediated via the calcitonin gene-related peptide in a rat

**DOI:** 10.1093/sleep/zsad332

**Published:** 2023-12-21

**Authors:** Ayano Katagiri, Saki Kishimoto, Yoshie Okamoto, Masaharu Yamada, Hitoshi Niwa, David A Bereiter, Takafumi Kato

**Affiliations:** Department of Oral Physiology, Osaka University Graduate School of Dentistry, Osaka, Japan; Department of Oral Physiology, Osaka University Graduate School of Dentistry, Osaka, Japan; Department of Dental Anesthesiology, Osaka University Graduate School of Dentistry, Osaka, Japan; Department of Oral Physiology, Osaka University Graduate School of Dentistry, Osaka, Japan; Department of Oral Physiology, Osaka University Graduate School of Dentistry, Osaka, Japan; Department of Dental Anesthesiology, Osaka University Graduate School of Dentistry, Osaka, Japan; Department of Dental Anesthesiology, Osaka University Graduate School of Dentistry, Osaka, Japan; Department of Diagnostic and Biological Sciences, University of Minnesota School of Dentistry, MN, USA; Department of Oral Physiology, Osaka University Graduate School of Dentistry, Osaka, Japan

**Keywords:** rats, calcitonin gene-related peptide, neurons, orofacial pain, hypoxia, obstructive sleep apnea

## Abstract

**Study Objectives:**

Obstructive sleep apnea, a significant hypoxic condition, may exacerbate several orofacial pain conditions. The study aims to define the involvement of calcitonin gene-related peptide (CGRP) in peripheral and central sensitization and in evoking orofacial mechanical allodynia under chronic intermittent hypoxia (CIH).

**Methods:**

Male rats were exposed to CIH. Orofacial mechanical allodynia was assessed using the eyeblink test and the two-bottle preference drinking test. The CGRP-immunoreactive neurons in the trigeminal ganglion (TG), CGRP-positive primary afferents projecting to laminae I–II of the trigeminal spinal subnucleus caudalis (Vc), and neural responses in the second-order neurons of the Vc were determined by immunohistochemistry. CGRP receptor antagonist was administrated in the TG.

**Results:**

CIH-induced ocular and intraoral mechanical allodynia. CGRP-immunoreactive neurons and activated satellite glial cells (SGCs) were significantly increased in the TG and the number of cFos-immunoreactive cells in laminae I–II of the Vc were significantly higher in CIH rats compared to normoxic rats. Local administration of the CGRP receptor antagonist in the TG of CIH rats attenuated orofacial mechanical allodynia; the number of CGRP-immunoreactive neurons and activated SGCs in the TG, and the density of CGRP-positive primary afferent terminals and the number of cFos-immunoreactive cells in laminae I–II of the Vc were significantly lower compared to vehicle-administrated CIH rats.

**Conclusions:**

An increase in CGRP in the TG induced by CIH, as well as orofacial mechanical allodynia and central sensitization of second-order neurons in the Vc, supported the notion that CGRP plays a critical role in CIH-induced orofacial mechanical allodynia.

## Graphical Abstract

**Graphical Abstract:**
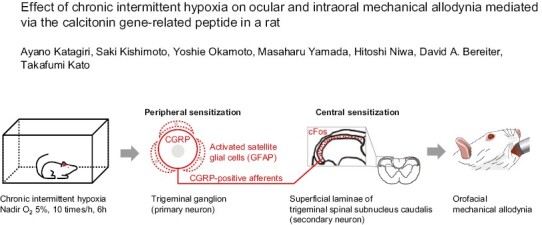

**Statement of Significance**

Obstructive sleep apnea (OSA) is characterized by recurrent nocturnal hypoxemia and may exacerbate several orofacial pain conditions. Male rats were exposed to chronic intermittent hypoxia (CIH) to establish the OSA model. CIH-induced orofacial mechanical allodynia and calcitonin gene-related peptide (CGRP) and cFos expression increased in the trigeminal ganglion (TG) and the trigeminal subnucleus caudaris (Vc) neurons, respectively. CGRP receptor antagonist administration in the TG of CIH rats significantly attenuated orofacial mechanical allodynia and decreased CGRP expression in the TG and the density of CGRP-positive primary afferent terminals in the Vc. Moreover, cFos expression in the Vc was suppressed. Peripheral increases in CGRP induced by CIH cause the central sensitization of the Vc neurons, which leads to orofacial mechanical allodynia.

## Introduction

Worldwide, approximately 425 million individuals experience moderate to severe obstructive sleep apnea (OSA) [1]. OSA is characterized by recurrent nocturnal hypoxemia and sleep fragmentation. The severity of sleep-related breathing cessation and desaturation substantially contributes to daytime sleepiness in patients with OSA [2]. Furthermore, 32% of adult patients with chronic pain also present with OSA [3], and experimental sleep fragmentation or deprivation enhances pain sensitivity [4, 5] and exacerbates spontaneous pain [6] in healthy individuals. Conversely, continuous positive airway pressure treatment attenuates pain sensitivity in patients with OSA [7]. Therefore, OSA, a significant hypoxic condition [8], is a critical risk factor for chronic pain [9].

OSA may exacerbate several orofacial pain conditions such as burning mouth syndrome [10], migraine [11], temporomandibular joint disorders [12], dry eye [13], and xerostomia [14]. Recent studies have indicated that oral appliance treatment for OSA may be beneficial for treating headaches in patients with OSA [15], whereas hypoxia can trigger migraine-like headaches in patients and healthy individuals [16]. Calcitonin gene-related peptide (CGRP) levels in venous blood, but not substance P, are elevated during acute migraine attacks [17] and after hypoxic challenge, akin to migraine attacks [18]. The density of CGRP-positive fibers innervating the oral mucosa is increased in patients with OSA [19], which suggests that CGRP is a critical factor causing orofacial pain in OSA-like conditions. Furthermore, animal studies have reported that CGRP expression in the trigeminal ganglion (TG) was significantly increased in models of neuropathic pain of the tongue and in ocular nociception after bright light stimulation [20, 21]. CGRP subsequently activates satellite glial cells (SGCs) that surround TG neurons, suggesting a significant role in neuron–glia interactions [21].

CIH exposure during the light period is a well-established animal model of sleep apnea. Severe hypoxic conditions and high cycle-frequency hypoxic episodes lead to significant pathological changes in the nervous system [22]. Previously, we reported that CIH (nadir O_2_ 5%, 10 times/h, 6 h/day, and 16 days) during the light period in rats caused transient ocular and intraoral hypersensitivity to capsaicin. Orofacial hypersensitivity was mediated via an increase of transient receptor potential vanilloid 1 (TRPV1)-immunoreactive (IR) neurons in the TG, TRPV1-positive primary afferents that project centrally to terminate in laminae I–II of the trigeminal spinal subnucleus caudalis (Vc), and capsaicin-responsive phosphorylated extracellular signal-regulated kinase-IR neurons in laminae I–II of the Vc [23]. Moreover, hypoxia (O_2_ level: 13%–15%) may directly activate Vc neurons [24]. A high percentage of CGRP-IR neurons in the TG co-express TRPV1 (70%), predominantly in the small-sized neurons [25, 26]. The vast majority of CGRP- and TRPV1-positive primary afferents project to laminae I–II of the Vc [25, 27], a brainstem region that integrates predominantly nociceptive information [28]. Collectively, previous studies suggested that peripheral CGRP is necessary for orofacial nociception under chronic intermittent hypoxia (CIH); however, the role of CGRP in activating second-order neurons in the Vc remains uncertain [24]. Therefore, the aim of this study was to better define the involvement of CGRP in peripheral and central sensitization and in evoking orofacial mechanical allodynia under CIH during the light period.

## Materials and Methods

The Animal Experimentation Committee at Osaka University Graduate School of Dentistry approved the protocol for this animal study (H29-032). All experimental procedures were performed in accordance with the Animal Research: Reporting of In Vivo Experiments guidelines 2.0 [29]. This study included 107 adult male Sprague–Dawley rats (body weight: 200–350 g; Japan SLC, Shizuoka, Japan). The data of four rats were excluded from the analysis because they could not acclimate to the behavioral test environment. The rats were housed in a light-controlled environment and climate (light period: 08:00–20:00; dark period: 20:00–08:00; temperature: 23°C  ± 0.5°C) for more than 1 week before the experiments. Water and food were provided ad libitum, even during the hypoxic protocol. Male sex has a positive effect on OSA prevalence [30]; therefore, only male rats were used in the present study. The operators were blinded to the treatment groups used in all experiments.

The details of the following procedures have been described previously: CIH, blood gas analysis, eyeblink tests, two-bottle preference drinking test [23], immunohistochemical analysis of the TG [23], immunohistochemical analysis from trigeminal spinal subnucleus interpolaris (Vi) to upper cervical spinal cord (C2) [23, 31–33], drug administration to the TG [21, 34], and measurement of tear/salivary volume [31, 32, 35].

### CIH protocol

**Figure 1.**
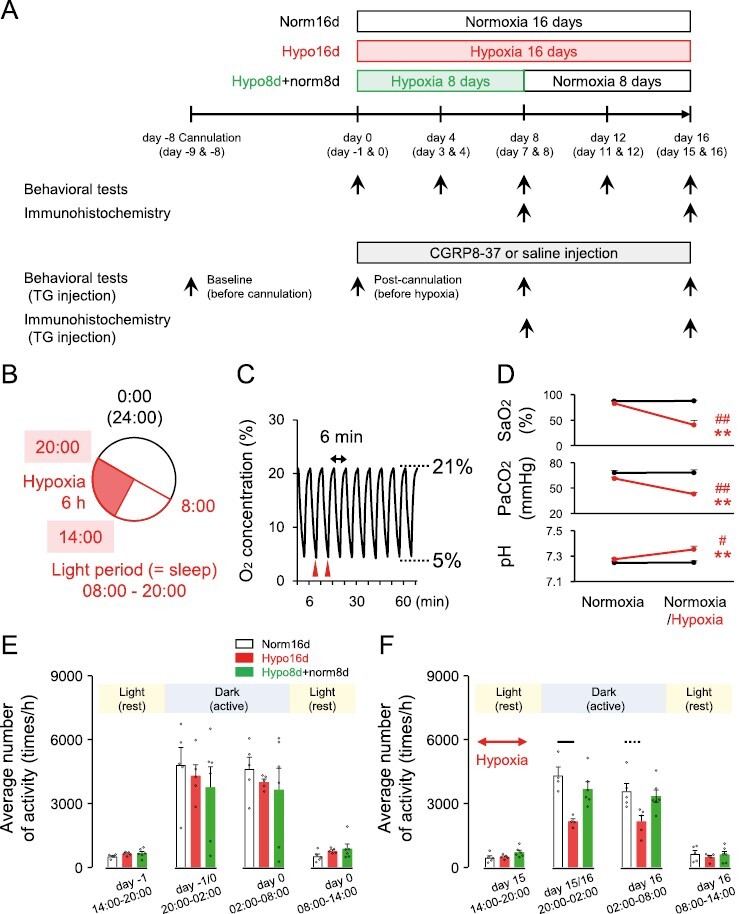
Experimental design and locomotor activity during chronic intermittent hypoxia (CIH). (A) Experimental design. Day 0, at 14:00–20:00, was the first day of intermittent hypoxia. Day 15 (Hypo16d group) or day 7 (Hypo8d + norm8d group) was the last day of intermittent hypoxia. Behavioral tests and collection of neural tissues for immunohistochemistry were performed from day 1 to day 16. Separate normoxia and intermittent hypoxia groups were administered a CGRP antagonist or saline in the TG for 8 or 16 days (gray box). (B) Daily protocol for intermittent hypoxia. Intermittent hypoxia: 14:00–20:00. Behavioral testing collection of neural tissues for immunohistochemistry: 08:00–14:00. (C) Actual O_2_ concentration during intermittent hypoxia. Red arrowheads indicate blood gas sampling. (D) Average SaO_2_ (upper panel), PaCO_2_ (middle panel), and pH (lower panel) at second and third cycles of intermittent hypoxia in naïve rats. Normoxia-normoxia: *n* = 5, normoxia-hypoxia: *n* = 5. **p < 0.01: (Normoxia-)Normoxia versus (Normoxia-)Hypoxia. # *p* < 0.05, # *p* < 0.01: pre versus post. (E) Locomotor activity (baseline, before chronic intermittent hypoxia). Norm16d: *n* = 5, Hypo16d: *n* = 5, Hypo8d + norm8d: *n* = 6. (F) Locomotor activity after chronic intermittent hypoxia. Dotted line: *p* < 0.05, black line: *p* < 0.01. Norm16d, normoxia 16 days; Hypo16d, hypoxia 16 days; Hypo8 + norm8, hypoxia 8 days and normoxia 8 days; SaO_2_, saturation of arterial oxygen; PaCO_2_, arterial partial pressure of carbon dioxide

The animals were divided into three groups: normoxia for 16 days (Norm16d group; control group), intermittent hypoxia for 16 days (Hypo16d group), and intermittent hypoxia for 8 days, followed by normoxia for 8 days (Hypo8d + norm8d group). Separate rats in the normoxia and intermittent hypoxia groups were administered a CGRP antagonist or saline (vehicle) in the TG for 8 or 16 days (Figure 1A). Behavioral tests were performed on days 0, 4, 8, 12, and 16. Two-bottle preference drinking tests were performed on two successive days: days −1 and 0; and on post-treatment days 3 and 4, days 7 and 8, days 11 and 12, and days 15 and 16. For immunohistochemical analyses, TG and lower brainstem tissue samples were collected on days 8 and 16. CIH was induced at 14:00 on day 0 and continued for 6 hours during the light period (sleep period for rats) (Figure 1B). Perfusion for immunohistochemical analysis and behavioral tests were conducted between 08:00 and 14:00. “Day 0” indicates the baseline before the first hypoxia protocol and “day 16” indicates the day after 16 applications of the hypoxia protocol from day 0 to day 15.

The CIH rats were exposed to intermittent hypoxic conditions (nadir O_2_ 5%, 3 minutes cycle, 14:00–20:00, SEVENz Planning Inc., Tokyo, Japan) (Figure 1B). Figure 1C depicts the monitoring of the oxygen concentration in the chamber.

### Blood gas analysis

The rats were anesthetized via intraperitoneal administration of saline solution mixed with medetomidine 0.375 mg/kg (ZENOAQ, Fukushima, Japan), midazolam 2.0 mg/kg (Sandoz, Tokyo, Japan), and butorphanol 2.5 mg/kg (M/M/B; Meiji Seika Pharma, Tokyo, Japan). Arterial blood was collected from the right femoral artery (100 µL). The first session was used to ascertain the pre-measurement values under normoxia. The second session was used to determine the post-measurement value under the normoxic or hypoxic condition, and the average value of the two samples in each session was calculated. Arterial blood was sampled at the lowest O_2_ concentration during the second and third hypoxia cycles (Figure 1C, red arrowheads). Saturation of arterial oxygen (SaO_2_), partial pressure of arterial carbon dioxide (PaCO_2_), and pH of arterial blood were analyzed (ABL800 FLEX, Radiometer).

### Behavioral tests

#### Locomotor activity.

Sleep fragmentation associated with OSA may cause daytime sleepiness [36]. Sleepiness can be indirectly observed to record locomotor activity [37]. Nano-Tags (18.8 × 14.2 × 7.1 mm^3^, 2.7 g; Kissei Comtec Co. Ltd., Nagano, Japan) were implanted under the skin of the back under 3% isoflurane anesthesia more than 10 days before recording. Nano-Tag methodology identifies sleep–wake states in animals [38]. The locomotor activity was recorded every 30 seconds and stored in Nano-Tag, and the data were transferred to the Nano-Tag Viewer program (Kissei Comtec Co. Ltd.) percutaneously at 11:00 under light isoflurane anesthesia. Two hours of data obtained from 11:00 to 13:00 were excluded from the analysis. The data were collected as the average of each hour. In the Nano-Tag device measurements, activity was defined as cross-count data, providing a count of the number of times the XYZ acceleration vector synthesized waveform crossed the threshold levels from the bottom (170/min) to the top (170/min) per recording interval. Locomotor activity was measured in group-housed rats (2–3 rats/cage) in each experimental group to prevent social separation stress.

#### Eyeblink tests.

Eyeblink tests were conducted to assess hypersensitivity and allodynia of the ocular region which are innervated by the ophthalmic branch of the TG. Eyeblinks, including partial and complete lid closures, were counted for 3 minutes after ocular instillation of hypertonic saline (0.15 M, 1.0 M, 2.5 M; 15 µL each) in the left eye. The same rats underwent mechanical stimulation via the application of von Frey filaments (0.008 g, 0.02 g, and 0.04 g) to the center of the left cornea. Three tests (each session: 20 seconds) were performed, and the average was calculated. Hypertonic saline instillation and mechanical stimuli were applied in ascending order of intensity. There was an interval of at least 30 minutes between each stimulus (i.e. hypertonic saline and von Frey filaments).

#### Two-bottle preference drinking test using a spout with mechanical stimulation.

A two-bottle preference drinking test using a spout with mechanical stimulation was conducted to detect mechanical hypersensitivity and allodynia of the tongue and lips. The two-bottle preference drinking test was administered for 2 hours after 22 hours of water deprivation [39]. During the test session, the rats had free access to two adjacent bottles containing distilled water with spouts (diameter = 6.0 mm), one with and the other without mechanical stimulation. The optical fibers (diameter = 0.5 mm; same material as used for von Frey filament test) were set parallel to the spout without any space, and the tip of each optical fiber was arranged randomly at 2.0–3.0 mm from the edge of the spout (Supplementary Video S1: normal spout; Supplementary Video S2: spout with mechanical stimulation). The positions of the two spouts were reversed daily to avoid positional preferences on the 2 consecutive testing days. Each bottle was weighed before and after the 2-hour drinking test session to measure the volume of fluid consumed. The consumption of distilled water from spouts with mechanical stimulation was quantified as the percentage of the total volume consumed during the 2 hours of the drinking test sessions on each test day for each rat. The average percentage of 2 successive days was calculated. Rats received water ad libitum from spouts without mechanical stimulation in their home cages during the periods when the drinking test was not being conducted.

#### Measurement of forelimb grip force.

Rats were held by their tail and passed over a wire mesh grid connected to a strain gauge, and the maximum forelimb grip force was determined from three trials at 10-minute intervals [40].

### Immunohistochemistry

The rats were anesthetized with pentobarbital sodium (80 mg/kg, intraperitoneal administration), followed by transcardial perfusion of 4% cold paraformaldehyde in 0.01 M PB (pH = 7.4) on days 8 or 16 after CIH (Figure 1A). The lower brainstem to the upper cervical spinal cord and TG, was removed. Some rats were used for immunohistochemical analysis after the behavioral tests.

#### Activated SGCs and CGRP expression in the TG.

Successive horizontal sections (10 µm) of the TG were collected at every twelfth section. The sections were incubated sequentially in primary antibodies (Table 1) for 48 hours at 4°C and secondary antibodies (Table 1) for 2 hours at 23°C.

**Table 1:** Antibody

Trigeminal ganglion				
Primay antibody	Dilution ratio	Cat#	Lot#	Company
Mouse anti-GFAP	1:800	MAB360	3698598	Millipore
Rabbit anti-CGRP	1:500	C8198	113M4760	Sigma-Aldrich
Secondary antibody	Dilution ratio	Cat#	Lot#	Company
Alexa Fluor 568 goat anti-mouse IgG	1:500	A11004	1698376	Invitrogen
Alexa Fluor 488 goat anti-rabbit IgG	1:500	A11008	1797971	Invitrogen
Trigeminal subnucleus caudalis				
Primay antibody	Dilution ratio	Cat#	Lot#	Company
Rabbit anti-cFos	1:5000	RPCA-cFos-AP	170-121416	EnCore
rabbit anti-CGRP	1:8000	C8198	113M4760	Sigma-Aldrich
Secondary antibody	Dilution ratio	Cat#	Lot#	Company
Biotinylated goat anti-rabbit antibody	1:1000	BA1000		Vector Laboratories

The sections were examined under a fluorescence microscope (BZ-X800; KEYENCE, Osaka, Japan). The number of neurons encircled by glial fibrillary acidic protein (GFAP)-IR SGCs over two-thirds of the perimeter of the somata, the number of CGRP-IR cells, and the number of immunonegative cells (BZ-H4; KEYENCE) were counted in four randomly selected sections in each slide. The number of neurons encircled by GFAP-IR SGCs or CGRP-IR neurons relative to all the neurons was calculated in the ophthalmic, maxillary, and mandibular branches (675 × 565 mm^2^) of the TG [41]. Immunopositive and immunonegative cells were defined by luminance range; immunopositive cells > 90 and immunonegative cells 50–90. The cell sizes of the CGRP-IR neurons were measured (WinROOF 2021; Mitani, Fukui, Japan) and divided into small- (<400 μm^2^), medium- (400–800 μm^2^), and large-sized neurons (>800 μm^2^).

#### Immunohistochemistry from Vi to C2.

Successive coronal 40 µm sections were collected in every fourth section (160-µm intervals). The sections were incubated sequentially in 0.3% H_2_O_2_ in methanol for 1 hour at 23°C, 5% normal goat serum with 0.3% Triton X-100 in 0.01 M PBS for 1 hour at 23°C, primary antibodies (Table 1) for 48 hours at 4°C, and secondary antibody (Table 1) for 2 hours at 23°C. The reaction products were visualized using the Vectastain Elite ABC Rabbit IgG Kit (PK-6101; Vector Laboratories, Newark, CA, USA) and the peroxidase substrate kit imPACT DAB (SK-4105; Vector Laboratories). The sections were examined under a microscope (BZ-X800; KEYENCE).

The immunopositive cells were categorized in 0.5-mm intervals, 2.0 mm rostral, and 6.5 mm caudal to the obex.

#### cFos expression from Vi to C2.

The mean number of cFos immunopositive cells per section were compared in the following regions to facilitate statistical comparison: Vi (+2.0 to + 1.0 mm relative to the obex), trigeminal spinal subnucleus interpolaris to trigeminal spinal subnucleus caudalis transition (Vi/Vc, +0.5 mm to −0.5 mm), main portion of the trigeminal spinal subnucleus caudalis (middle-Vc, −1.0 mm to −4.0 mm), and caudal portion of the trigeminal spinal subnucleus caudalis to C2 transition (caudal-Vc/C2, −4.5 mm to −6.5 mm). The cFos-IR cells in the middle-Vc and caudal-Vc/C2 regions were counted separately in the superficial (I–II) and deeper laminae (III–V) and included the full dorsoventral extent of the Vc innervated by the ophthalmic, maxillary, and mandibular branches of the trigeminal nerve [42]. The cFos-IR cells were counted bilaterally and summated in each section for statistical analysis.

#### Density of CGRP-positive afferent terminals in the Vc.

The density of CGRP-positive central terminals in the Vc, which identifies primary afferents projecting to laminae I–II of the Vc, was analyzed ipsilaterally in laminae I–II of the Vc, 1.0 (P1.0), 3.0 (P3.0), and 5.0 (P5.0) mm posterior to the obex. The density was defined with a luminance range (0–130) using an imaging analysis system (BZ-H4; KEYENCE). Measurements were obtained from 100 × 100 µm^2^ regions of laminae I–II of the Vc and the mean value was calculated in each rostro-caudal level.

### CGRP8-37 administration to the TG

The rats were anesthetized by M/M/B and placed in a stereotaxic apparatus. A guide cannula was implanted into a hole on the skull (diameter = 1 mm, 2.7 mm from the sutura sagittalis and 2.9 mm anterior to the lambda bilaterally) 9.0 mm below the skull surface and fixed. The rats were allowed to recover for more than 1 week before the commencement of the experiments.

The CGRP receptor antagonist CGRP8-37 (0.2 nmol in 1.0 μL/day; Cat# 1169, batch# 27, CAS# 129121-73-9; Tocris Bio-Techne Japan, Tokyo, Japan) [43] or isotonic saline (1.0 μL/day, vehicle) was administrated into the TG for 16 successive days (days 0 to 15, once a day) through the guide cannula between the behavioral tests and CIH under 2% isoflurane anesthesia. Isotonic saline administration into the TG of naïve rats has been shown to have no effects on nociceptive thresholds and CGRP expression in the TG [44]. There were no significant differences between Norm16d rats without any administration into the TG and CGRP8-37-administrated Norm16d rats in all behavioral tests and immunohistochemistry experiments.

### Measurement of tear and salivary volume

The respective volumes of tears and saliva were measured under pentobarbital sodium anesthesia (80 mg/kg, intraperitoneal) before perfusion. The spontaneous tear volume of both eyes was measured for 2 minutes by the increase in the wet length of a phenol red thread (ZONE-QUICK; AYUMI Pharmaceutical Co., Tokyo, Japan) [31, 32]. The average of the right and left eyes was calculated. Spontaneous salivary volume was measured for 1 minute by the increase in the wet length of a phenol red thread (ZONE-QUICK; AYUMI Pharmaceutical Co.) placed gently in the sublingual area [35]. Food and water were withheld for 3 hours before spontaneous salivary volume measurement.

### Statistical analysis

The Kruskal–Wallis test, followed by Dunn’s test, was used to analyze and compare locomotor activity, tear volume, salivary volume, and immunohistochemistry data from each group. A two-way analysis of variance, followed by the Bonferroni test, was used to analyze and compare, at each time point to assess group differences and baseline directly (“day 0” or “day -1 & 0”) in blood gas, body weight, grip force, and all behavioral tests (GraphPad Prism version. 7.02; GraphPad Software Inc., La Jolla, CA, USA). The chi-squared test was used to analyze and compare the ratio of CGRP-IR cells among the three different neuron-size groups. The data in the text and figures are presented as mean ± SEM. The significance level was set at *p* < 0.05. A sample size of *n* = 5 per treatment group would provide 80% statistical power at *p* < 0.05. The actual numbers and *p*-values in each graph are summarized in Supplementary Table S1-S9.

## Results

### General condition during the CIH protocol

SaO_2_ and PaCO_2_ decreased significantly in rats exposed to hypoxia compared to those under normoxia. The pH of arterial blood was significantly higher under hypoxic conditions than under normoxic conditions (Figure 1D).

Locomotor activity did not differ significantly among the Norm16d, Hypo16d, and Hypo8d + norm8d rats during the light (rest) period, even after 6 hours of hypoxia. However, locomotor activity was significantly reduced in Hypo16d rats compared to the Norm16d rats and recovered in Hypo8d + norm8d rats during the dark (active) period (Figure 1E: before CIH protocol, Figure 1F: on days 15 and 16 after CIH protocol). Body weight was significantly lower in Hypo16d and Hypo8 + norm8d rats than in Norm16d rats (Supplementary Figure S1A). Body weight in Hypo8 + norm8d rats recovered slightly after 8 days of hypoxia compared to that in Hypo16d rats, whereas no significant recovery was observed compared to that in Norm16d rats. Grip force was similar for Norm16d, Hypo16d, and Hypo8d + norm8d rats (Supplementary Figure S1B, the same rats are referred to in Supplementary Figure S1A). These results indicated that CIH affected body weight and locomotor activity but not muscular strength.

### Tear volume and eye hypersensitivity to osmotic pressure and mechanical stimulation

**Figure 2.**
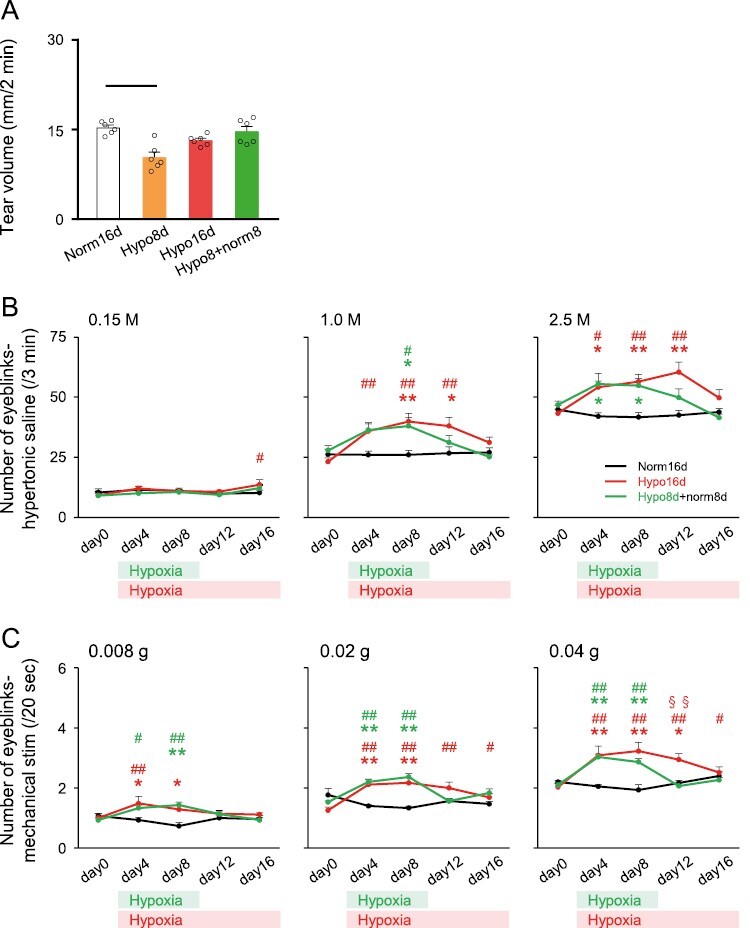
Tear volume and eyeblink responses to hypertonic saline and mechanical stimulation of the eye. (A) Spontaneous tear volume. Norm16d: *n* = 6, Hypo8d: *n* = 6, Hypo16d: *n* = 6, Hypo8d + norm8d: *n* = 6. Black line: *p* < 0.01. (B) Number of eyeblinks evoked by ocular instillation of hypertonic saline (left graph: 0.15 M, central graph: 1.0 M, and right graph: 2.5 M). Norm16d: *n* = 6, Hypo16d: *n* = 7, Hypo8d + norm8d: *n* = 6. * *p* < 0.05, ** *p* < 0.01: versus Norm16d. # *p* < 0.05, ## *p* < 0.01: versus day 0. (C) Number of eyeblinks evoked by corneal mechanical stimulation (left graph: 0.008 g, central graph: 0.02 g, and right graph: 0.04 g). The green and red crossbars at the bottom of the graph indicate periods of intermittent hypoxia in the Hypo8d + norm8d and Hypo16d groups, respectively. Norm16d: *n* = 6, Hypo16d: *n* = 7, Hypo8d + norm8d: *n* = 6. * *p* < 0.05, ** *p* < 0.01: versus Norm16d. # *p* < 0.05, ## *p* < 0.01: versus day 0. §§ *p* < 0.01: versus Hypo8d + norm8d. Norm16d, normoxia 16 days; Hypo16d, hypoxia 16 days; Hypo8 + norm8, hypoxia 8 days and normoxia 8 days.

Spontaneous tear volume declined significantly under hypoxic conditions on day 8 compared to Norm16d rats. On day 8, the spontaneous tear volume recovered in rats subjected to normoxia after hypoxia for 8 days (Hypo8 + norm8 on day 16) (Figure 2A).

There was no significant difference in the number of eyeblinks evoked by 0.15 M NaCl (concentration ≈ isotonic saline) instillation between Norm16d, Hypo16d, and Hypo8d + norm8d rats. However, the number of eyeblinks evoked by hypertonic saline (1.0 M NaCl, 2.5 M NaCl) was significantly higher in rats exposed to CIH compared to that in Norm16d rats (i.e. hyperalgesia), which subsequently recovered with/without CIH for 16 days (Figure 2B).

The number of mechanical stimulation-evoked eyeblinks was significantly higher under CIH exposure compared to that in Norm16d rats, which subsequently recovered with/without CIH for 16 days (Figure 2C). The results indicated that CIH transiently reduced spontaneous tear volume and that corneal sensitivity to osmotic pressure and mechanical stimulation due to CIH were markedly increased.

### Salivary volume and intraoral mechanical allodynia

**Figure 3.**
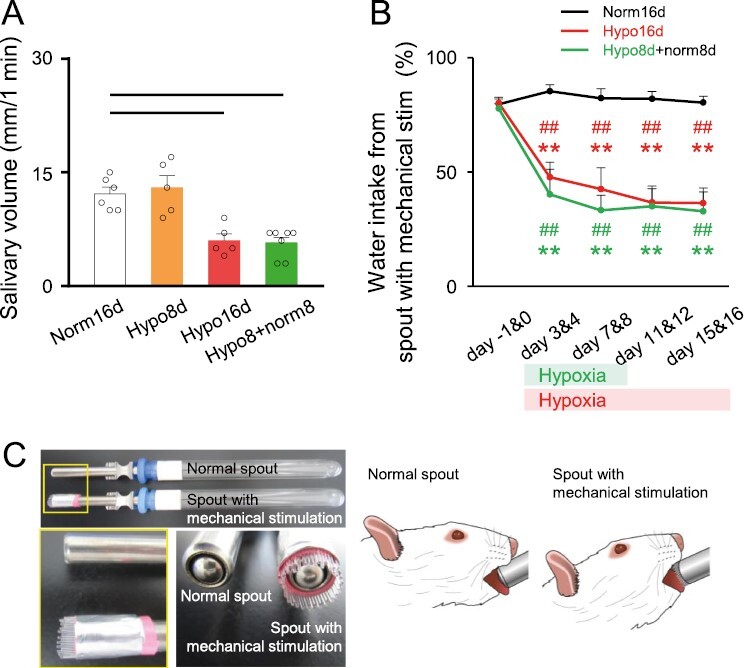
Salivary volume and sensitivity to intraoral mechanical stimulation. (A) Spontaneous salivary volume. Norm16d: *n* = 7, Hypo8d: *n* = 6, Hypo16d: *n* = 5, Hypo8d + norm8d: *n* = 8. Dotted line: *p* < 0.05. (B) The ratio of water intake from the spout with mechanical stimulation. Norm16d: *n* = 8, Hypo16d: *n* = 7, Hypo8d + norm8d: *n* = 6. ** *p* < 0.01: versus Norm16d. ## *p* < 0.01: versus day 0. (C) Normal spout and spout with mechanical stimulation for two-bottle preference drinking test. (Supplementary illustrations: high-resolution illustrations of Figure 3C, Supplementary Video S1: drinking water from a normal spout, Supplementary Video S2: drinking water from a spout with mechanical stimulation [optical fibers]). Norm16d, normoxia 16 days; Hypo16d, hypoxia 16 days; Hypo8 + norm8, hypoxia 8 days and normoxia 8 days.

The spontaneous salivary volume declined significantly after 16 days of CIH treatment. CIH for 8 days did not affect salivary volume; however, on day 8, the spontaneous salivary volume in rats subjected to normoxic conditions following hypoxia for 8 days (Hypo8 + norm8 on day 16) was significantly lower than that of Norm16d rats (Figure 3A).

The ratio of water intake from the bottle with mechanical stimulation to the total volume consumed was significantly lower during the CIH protocol and on day 8 under normoxic conditions following hypoxia for 8 days (Hypo8 + norm8 on day 16) compared to that in Norm16d rats. No recovery from intraoral mechanical allodynia was observed (Figure 3B). There was no significant difference in the total water intake volume consumed during 2 hours of the drinking test sessions among Norm16d, Hypo16d, and Hypo8 + norm8d groups (data not shown). Therefore, CIH did not affect drinking function. These results indicated that CIH reduced spontaneous salivary volume and induced persistent intraoral mechanical allodynia. The normal spout and spout with mechanical stimulation for the two-bottle preference drinking test to investigate oral mechanical allodynia is illustrated in Figure 3C and Supplementary Video S1 and S2.

### Satellite glial activation and CGRP expression in the TG

**Figure 4.**
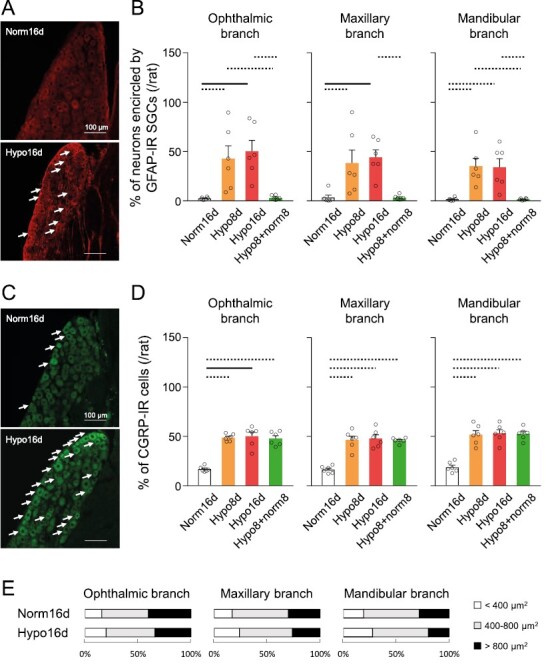
Satellite glial cells activation and CGRP expression in the TG. (A) Photomicrographs of GFAP-IR SGCs in the mandibular branch of the TG. The arrows indicate GFAP-IR SGCs. Scale bar = 100 µm. (B) Percentage of neurons encircled by GFAP-IR SGCs in the ophthalmic, maxillary, and mandibular nerve branches of the TG. Norm16d: *n* = 6, Hypo8d: *n* = 6, Hypo16d: *n* = 6, Hypo8d + norm8d: *n* = 6. Dotted line: *p* < 0.05, black line: *p* < 0.01. (C) Photomicrographs of CGRP-IR cells in the mandibular branch of the TG. The arrows indicate CGRP-IR cells. Scale bar = 100 µm. (D) Percentage of CGRP-IR cells in the ophthalmic, maxillary, and mandibular nerve branches of the TG. Norm16d: *n* = 6, Hypo8d: *n* = 6, Hypo16d: *n* = 6, Hypo8d + norm8d: *n* = 6. Dotted line: *p* < 0.05, black line: *p* < 0.01. (E) Ratio of CGRP-IR cells of different sizes in the ophthalmic, maxillary, and mandibular nerve branches of the TG. Norm16d, normoxia 16 days; Hypo16d, hypoxia 16 days; Hypo8 + norm8, hypoxia 8 days and normoxia 8 days; TG, trigeminal ganglion; GFAP, glial fibrillary acidic protein; SGCs, Satellite glial cells; CGRP, calcitonin gene-related peptide.

CIH resulted in enhanced satellite glial activation in the TG (Figure 4A). The ratio of neurons encircled by GFAP-IR SGCs in all branches of the TG in CIH rats was significantly higher under CIH compared to Norm16d rats and was reversed on day 8 under normoxia following hypoxia for 8 days (Hypo8 + norm8 on day 16) (Figure 4B).

CIH resulted in enhanced CGRP expression in the TG (Figure 4C). The ratio of CGRP-IR cells in all branches of the TG in CIH rats was significantly higher during CIH than that under Norm16d, and, unlike satellite glial activation, did not recover on day 8 under normoxia following hypoxia for 8 days (Hypo8 + norm8 on day 16) (Figure 4D, this figure references the rats mentioned in Figure 4B). No significant change was observed in the ratios of small-, medium-, and large-sized CGRP-IR cells after 16 days of CIH (Figure 4E). The results indicated that the time-course change in CGRP expression in the TG correlated well with that of intraoral mechanical allodynia during the CIH protocol. However, there was no apparent change in the cell size distribution of the CGRP-IR TG neurons.

### cFos expression from Vi to C2

**Figure 5.**
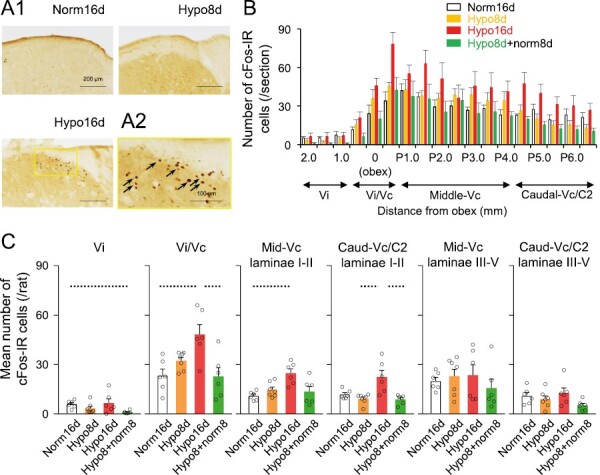
cFos expression in the caudal trigeminal brainstem. (A1) Photomicrograph of cFos-IR cells in the Vc. Scale bar = 200 µm. (A2) High magnification of the yellow-lined box in Hypo16d of Figure 5A1 facilitates visualization of the cFos-IR cells. Scale bar = 100 µm. (B) Distribution of cFos-IR cells throughout the rostro-caudal region from Vi to caudal-Vc/C2. The middle-Vc and caudal-Vc/C2 include laminae I–II and laminae III–V in this distribution graph. Norm16d: *n* = 6, Hypo8d: *n* = 7, Hypo16d: *n* = 6, Hypo8d + norm8d: *n* = 6. (C) Mean number of cFos-IR cells in the Vi, Vi/Vc, middle-Vc, and Caudal-Vc/C2. Dotted line: *p* < 0.05. Norm16d, normoxia 16 days; Hypo16d, hypoxia 16 days; Hypo8 + norm8, hypoxia 8 days and normoxia 8 days; Vi, trigeminal spinal subnucleus interpolaris; Vc, trigeminal spinal subnucleus caudalis; Vi/Vc, trigeminal subnucleus interpolaris/caudalis transition; Mid, middle; Caud, caudal; C2, upper cervical spinal cord; P, posterior from obex.

CIH resulted in an increase in the number of cFos-IR cells from the Vi/Vc to caudal-Vc/C2 on day 16 (Figure 5, A1–2 and B). The number of cFos-IR cells in the Vi/Vc, laminae I–II of middle-Vc, and laminae I–II of caudal-Vc/C2 was significantly higher under hypoxia on day 16 compared to normoxia on day 16, hypoxia on day 8, or Hypo8 + norm8 on day 16 rats (Figure 5C). There were no significant differences in laminae III–V (deep) of the middle-Vc and caudal-Vc/C2 among all groups (Figure 5C). These results indicated that CIH for 16 days enhanced neuronal activity, especially in laminae I–II of the Vc, which is closely associated with orofacial nociceptive processing.

### Effects of CGRP receptor inhibition in the TG on mechanical allodynia

**Figure 6.**
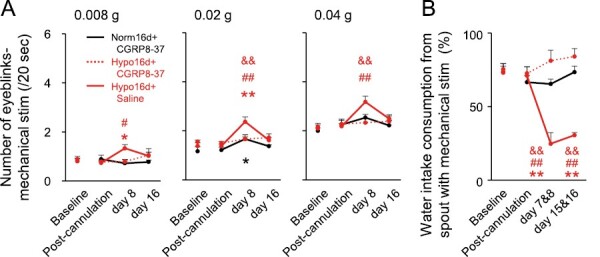
Effects of TG administration of CGRP8-37 on ocular and intraoral mechanical allodynia. (A) Number of eyeblinks evoked by mechanical stimulation (0.008 g, 0.02 g, 0.04 g). Norm16d + CGRP8-37: *n* = 6, Hypo16d + CGRP8-37: *n* = 6, Hypo16d + saline: *n* = 7. * *p* < 0.05, ** *p* < 0.01: versus Norm16d + CGRP8-37. # *p* < 0.05, ## *p* < 0.01: versus baseline. && p < 0.01: versus Hypo16d + CGRP8-37. (B) The ratio of water intake from the spout with mechanical stimulation. Norm16d + CGRP8-37: *n* = 6, Hypo16d + CGRP8-37: *n* = 5, Hypo16d + saline: *n* = 7. ** *p* < 0.01: versus Norm16d + CGRP8-37. ## *p* < 0.01: versus baseline. && *p* < 0.01: versus Hypo16d + CGRP8-37. Norm16d, normoxia 16 days; Hypo16d, hypoxia 16 days; TG, trigeminal ganglion; CGRP, calcitonin gene-related peptide.

The number of eyeblinks evoked by mechanical stimulation to the cornea was significantly lower in CGRP8-37-administered Hypo16d rats than for saline-treated Hypo16d rats (Figure 6A). The ratio of water intake from the bottle with mechanical stimulation was significantly higher in CGRP8-37-administered Hypo16d rats than in saline-administered Hypo16d rats (Figure 6B). The results indicated that CGRP receptor inhibition in the TG attenuated orofacial mechanical allodynia.

### Effects of CGRP receptor inhibition in the TG

**Figure 7.**
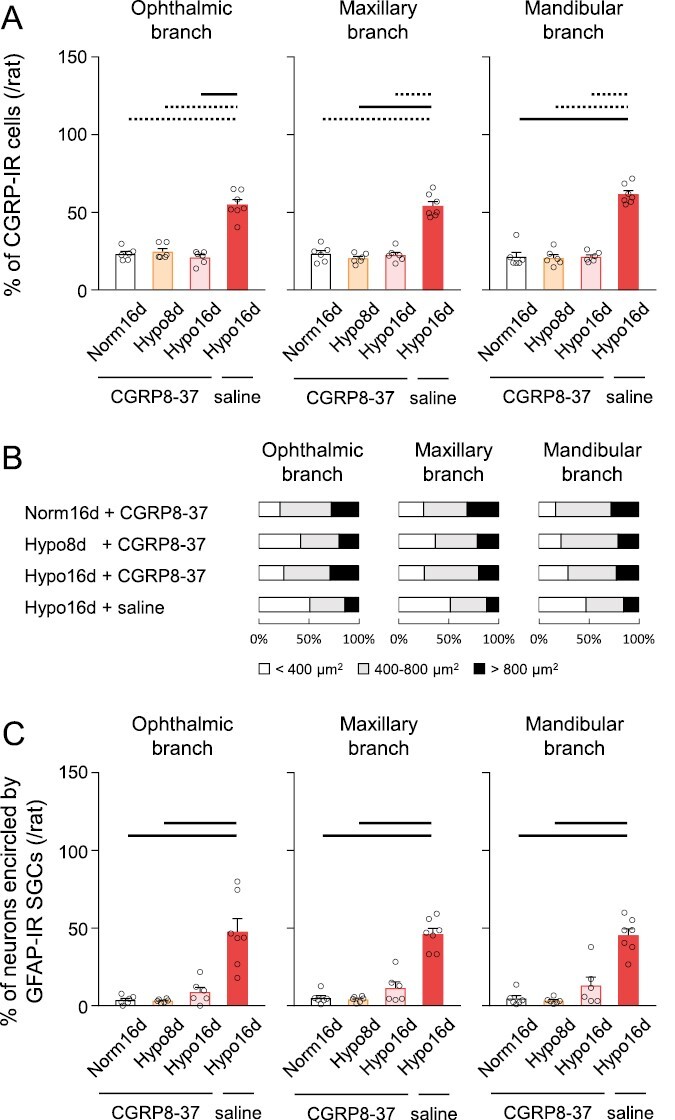
Effects of TG administration of CGRP8-37 on CGRP and GFAP expression in the TG. (A) Percentage of CGRP-IR cells in the ophthalmic, maxillary, and mandibular nerve branches of the TG. Norm16d + CGRP8-37: *n* = 6, Hypo8d + CGRP8-37: *n* = 6, Hypo16d + CGRP8-37: *n* = 6, Hypo16d + saline: *n* = 7. Dotted line: *p* < 0.05, black line: *p* < 0.01. (B) Ratio of CGRP-IR cells of different sizes in the ophthalmic, maxillary, and mandibular nerve branches of the TG. (C) Percentage of neurons encircled by GFAP-IR SGCs in the ophthalmic, maxillary, and mandibular nerve branches of the TG. Norm16d + CGRP8-37: *n* = 6, Hypo8d + CGRP8-37: *n* = 6, Hypo16d + CGRP8-37: *n* = 6, Hypo16d + saline: *n* = 7. Black line: *p* < 0.01. Norm16d, normoxia 16 days; Hypo8d, hypoxia 8 days; Hypo16d, hypoxia 16 days; TG, trigeminal ganglion; CGRP, calcitonin gene-related peptide; GFAP, glial fibrillary acidic protein; SGCs, satellite glial cells.

Local microinjection of CGRP8-37 into the TG significantly suppressed the increase in CIH-induced CGRP expression in all branches of the TG compared to saline-administered hypoxic rats. No significant changes were observed in the ratios of small-, medium-, and large-sized CGRP-IR cells after CIH and CGRP8-37 administration (Figure 7, A and B). The ratio of neurons encircled by GFAP-IR SGCs in all branches of the TG in CIH rats also was significantly lower in CGRP8-37-administered CIH rats than in saline-treated CIH rats (Figure 7C). There was no significant difference in CGRP and GFAP expression in all branches of the TG between Norm16d (Figure 4, B and D) and Norm16d + CGRP8-37 (Figure 7, A and C), and between Hypo16d (Figure 4, B and D) and Hypo16d + saline (Figure 7, A and C). These data indicated that local microinjection into the TG itself might not affect CGRP and GFAP expression in the TG [44]. Together with the behavioral data, these results indicated that CGRP upregulation in the TG played a critical role in developing ocular and intraoral mechanical allodynia under CIH.

### Effects of CGRP receptor inhibition in the TG on the central terminals of CGRP-positive primary afferents in the Vc and cFos expression from Vi to C2

**Figure 8.**
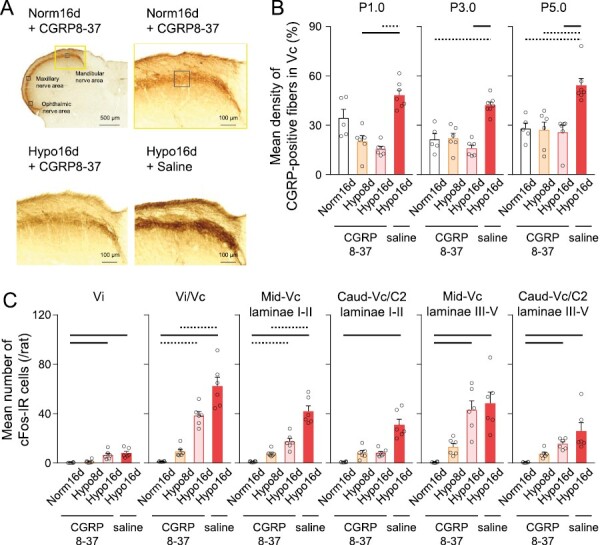
Effects of TG administration of CGRP8-37 on CGRP-positive primary afferents and cFos expression in the Vc. (A) Photomicrographs of CGRP-positive primary afferent terminals in the Vc. The black-lined boxes in Norm16d + CGRP8-37 indicate the ophthalmic, maxillary, and mandibular nerve areas in the Vc. Scale bar = 500 µm. High magnification of the yellow-lined box in Norm16d + CGRP8-37 facilitates visualization of the CGRP-positive primary afferent terminals. Scale bar = 100 µm. (B) Mean density of CGRP-positive primary afferent terminals in the 1.0 (P1.0), 3.0 (P3.0), and 5.0 (P5.0) mm posterior to the obex.Norm16d + CGRP8-37: *n* = 5, Hypo8d + CGRP8-37: *n* = 6, Hypo16d + CGRP8-37: *n* = 6, Hypo16d + saline: *n* = 7. Dotted line: *p* < 0.05, black line: *p* < 0.01. (C) Mean number of cFos-IR cells in the Vi, Vi/Vc, middle-Vc, and Caudal-Vc/C2. Norm16d + CGRP8-37: *n* = 6, Hypo8d + CGRP8-37: *n* = 6, Hypo16d + CGRP8-37: *n* = 6, Hypo16d + saline: *n* = 6. Dotted line: *p* < 0.05, black line: *p* < 0.01. Norm16d, normoxia 16 days; Hypo8d, hypoxia 8 days; Hypo16d, hypoxia 16 days; Vi, trigeminal spinal subnucleus interpolaris; Vc, trigeminal spinal subnucleus caudalis; Vi/Vc, trigeminal subnucleus interpolaris/caudalis transition; Mid, middle; Caud, caudal; C2, upper cervical spinal cord; P, posterior from obex.

Microinjection of CGRP8-37 into the TG suppressed the density of CGRP-positive primary afferents that project to laminae I–II of the Vc in hypoxic rats (Figure 8, A and B). The density of CGRP-positive primary afferent terminals in laminae I–II of the Vc was significantly higher in the P3.0 and P5.0 rostrocaudal levels in saline (vehicle of CGRP8-37)-administered hypoxic rats compared with CGRP8-37-administered normoxic rats and was significantly lower in CGRP8-37-administered hypoxic rats compared with saline-administered hypoxic rats (Figure 8B). Furthermore, TG administration of CGRP8-37 suppressed the increase in the number of cFos-IR in laminae I–II of the caudal-Vc/C2 of CGRP8-37-administered hypoxic rats (Figure 8C). These results indicated that CGRP upregulation in peripheral neurons may cause the sensitization of second-order neurons in the caudal Vc in CIH rats.

## Discussion

This study was designed to better define the role of CGRP in peripheral and central sensitization and in evoking orofacial mechanical allodynia in rats exposed to CIH during the light period. Our results indicated that CIH induced the following: (1) orofacial mechanical allodynia, (2) an increase in the number of activated SGCs and CGRP-IR cells in the TG, and (3) an increase in the number of cFos-IR neurons in the Vi/Vc and laminae I–II of the Vc. Furthermore, (4) local TG microinjection of CGRP8-37 in CIH rats suppressed mechanical allodynia, reduced CGRP and GFAP expression in the TG, and reduced the density of CGRP-positive primary afferent terminals and cFos-IR neurons in laminae I–II of the caudal-Vc. These results were consistent with the hypothesis that CGRP is necessary for peripheral and central sensitization of second-order neurons in the caudal-Vc neurons in CIH-induced orofacial mechanical allodynia.

### CIH during the light period

Although it has been reported that chronic non-rapid eye movement sleep deprivation in healthy mice progressively develops increased sleepiness, locomotor activity was conserved [45]. In contrast, chronic upper airway obstruction enhances orexin secretion which leads to chronic sleep loss and excessive active (dark) period sleepiness accompanied by lower locomotor activity in rats [37]. In this study, locomotor activity during the dark (active) period was significantly decreased in CIH rats. CIH may exert more substantial effects on the characteristics of daytime sleepiness in patients with OSA compared to sleep fragmentation or sleep loss [2]. However, in a migraine model, mice also showed reduced locomotor activity accompanied by increased CGRP expression in the TG [46]. Thus, the possibility that nociceptive states can reduce locomotor activity in CIH-exposed rats cannot be excluded.

Obesity is a major risk factor for OSA, whereas treatment with continuous positive airway pressure therapy leads to weight gain [47]. It is possible that the weight loss seen in CIH rats on days 8 and 16 in the present study was induced by the inhibition of food intake during the 6-hour hypoxic protocol [48] and lower locomotor activity in the dark (active) period [37].

### Mechanical allodynia under CIH

CIH increases pain in patients with OSA [49] and decreases nociceptive thresholds in animals [23]. The drinking test using capsaicin solution was adopted to detect intraoral capsaicin aversion, a test that mainly involves C-fiber activation [50–52]. Intraoral sensitivity to capsaicin in CIH rats was also detected using the two-bottle preference drinking test in our previous study [23]. Pinch stimulation of the tongue is a well-established method to detect mechanical allodynia in several tongue pain models [21, 34, 35, 44, 53–55]; however, pinch stimulation of the tongue can only be applied under isoflurane anesthesia. In this study, we established a novel method using a two-bottle preference drinking test with one spout having mechanical stimuli to assess intraoral mechanical allodynia in freely moving animals. As a preliminary experiment, we confirmed that this novel method can detect mechanical allodynia in an established model of neuropathic tongue pain (< 30%) [21, 34, 44] and a model of tongue inflammatory pain (< 30%) [53] which reduces the threshold for mechanical stimulation to the tongue (unpublished data). Therefore, this method can aid in the detection of spontaneous nociceptive behavior in intraoral structures in animals.

### Effects of CGRP on peripheral sensitization

TG neurons are classified as small, medium-, and large-sized cells, each giving rise to C, Aδ, and Aβ, afferent fibers respectively, and C and Aδ fibers normally mediate noxious responses [56]. CGRP is synthesized and released by small- to medium-sized TG neurons [57]. CGRP receptors are predominant in the medium- and large-sized TG neurons and SGCs [58]. In a rat model of tongue neuropathic pain, the number of CGRP-IR neurons increased accompanied by an increase in the ratio of large-sized CGRP-IR TG neurons. Moreover, CGRP released by TG neurons activated SGCs and enhanced TG neuronal activity via CGRP receptors, which indicated that tongue neuropathic pain was accompanied by changes in the cell size distribution of CGRP-IR TG neurons [21] similar to CGRP-IR dorsal root ganglion neurons in sciatic nerve-injured animals [59]. Moreover, a subpopulation of axotomized large-sized DRG neurons changes to synthesize CGRP de novo and release it [59–61]. Thus, Aβ afferent fibers may drive CGRP-sensitive central nervous system which is related to nociceptive signaling under neuropathic pain [62]. However, changes in the cell size distribution of CGRP-IR TG neurons were not observed in CIH rats in this study. Therefore, the upregulation of CGRP in the TG by CIH may be caused by different mechanisms of nerve injury and the involvement of Aβ afferent fibers in mechanical allodynia under CIH is limited.

The mechanisms for CGRP upregulation in the TG probably involve interactions between TRP channels and several factors increased by hypoxia such as hypoxia-inducible factor (HIF)-1α, reactive oxygen species (ROS), and nitric oxide (NO). HIF-1α is stable under hypoxia [63], and chronic hypoxemia in patients with OSA induces a sustained elevation of HIF-1α protein levels [64] and excessive ROS formation [65]. Animal studies found that CIH also leads to oxidative stress that involves upregulation of HIF-1α and ROS production [66–69] and induces an oxidative environment that enhances nociceptive transduction and transmission. HIF-1α acts as a transcriptional regulator of the molecular hypoxia response and sensitizes TRP ankyrin 1 (TRPA1) [70] and TRPV1 [71]. ROS may also trigger TRPA1 [72] and TRPV1 activation [73]. CGRP is released after neuronal depolarization by Ca^2+^-dependent exocytosis. The activation of Ca^2+^ ion channels (i.e. TRPV1 and TRPA1) enhances CGRP release from sensory neurons [72, 73]. These findings suggest that CIH upregulates CGRP in the TG by activating Ca^2+^-dependent ion channels. NO also may be involved in CGRP upregulation in the TG by acting through neuron–glia communication. Furthermore, hypoxia activates NO production in sensory neurons [74], which increases CGRP release in the TG [75] via activation of TRPV1 and TRPA1 [76]. Furthermore, CGRP released by TG neurons can induce NO production in SGCs [77], and activation of SGCs via CGRP receptors [21] could result in greater CGRP expression [77], directly activating adjacent neurons that express CGRP receptors [78]. Thus, CGRP-mediated positive feedback in neuron–glia and neuron–neuron interactions is thought to play a significant role in peripheral sensitization. In contrast, NO-mediated elevations in CGRP are not caused by an increase in CGRP mRNA [79]. This may be responsible for the lack of changes in the cell size distribution of CGRP-IR neurons in the TG, unlike TRPV1-IR neurons [23].

The time course of increased expression of CGRP is persistent, whereas that of TRPV1 [23] and SGC activation is more transient. As mentioned above, CGRP release is promoted by the activation of the TRP channel family [76]. TRPV1 in the TG is primarily activated as an early response, whereas TRPA1 is activated as a late response to orofacial noxious stimulation [80]. Because several factors activate SGCs, and not CGRP alone [81], this suggests that the transient increase in SGC activation is an active process. Increased CGRP expression in the TG was not persistent in tongue neuropathic pain models [21, 34]. Prolonged hypoxia was sufficient to increase CGRP plasma levels in patients with migraine [18]. Therefore, it can be inferred that hypoxia has a substantial impact on CGRP upregulation in the TG.

### Involvement of CGRP-positive primary afferents in central sensitization in the Vc

Peripheral mechanisms are likely driving forces for central sensitization and chronic pain [82]. CGRP levels in the primary afferent terminals of the spinal dorsal horn are elevated in animal models of chronic pain [83]. In other models, CGRP released from the central terminals of TG neurons projecting to laminae I–II of the Vc activates second-order neurons in the Vc via CGRP receptors [78], and intrathecally administered CGRP induces mechanical hypersensitivity in a dose-dependent manner [84]. The decrease in density of CGRP-positive primary afferents projecting to laminae I–II of the Vc by CGRP8-37 administration into the TG under CIH was associated with suppression of the number of cFos-IR neurons in laminae I–II of the caudal-Vc. These data indicated that the effects of CGRP released from the terminals of TG neurons onto second-order neurons in the Vc were necessary for the orofacial mechanical allodynia under CIH. A limitation of our study was that only male rats were used because the prevalence of OSA is reported to be higher in males [30]; however, CGRP may promote obvious nociceptive plasticity in secondary neurons in female animals [84]. Considering that burning mouth syndrome [10] and migraine [11] are more prevalent in female patients with OSA, further investigations into the role of CGRP as a factor in hypoxia-induced nociceptive responses in female animals are required. Furthermore, we cannot exclude that second-order neurons in laminae I–II of the Vc were activated by other peripheral mechanisms, such as NO produced by hypoxia [74], which is known to increase the activity of Vc neurons [85], or the possibility of direct activation of Vc neurons by hypoxia [24].

### Peripheral dryness

Inhibition of CGRP-IR neurons in the TG and CGRP-positive primary afferents in laminae I–II of the Vc attenuated orofacial mechanical allodynia, and the number of cFos-IR neurons in laminae I–II of the Vc was reduced in CIH-treated rats. Some trigeminal primary afferents (e.g. CGRP-positive [86] and TRPV1-positive [87] primary afferents) also directly project to the lateral parabrachial nucleus (LPBN). The LPBN plays a pivotal role in orofacial mechanical allodynia [33] and affective pain [88]. CIH-exposed rats exhibited symptoms of dry eye and dry mouth, consistent with evidence that hypoxia elevates sympathetic activity [24]. Preclinical studies have suggested that dryness and mechanical allodynia involve peripheral [31, 35, 55] and central sensitization [32, 54]. This is consistent with the suppression of ocular nociceptive responses associated with the recovery of tear fluid. It is not yet known if the monosynaptic circuits from the TG to the LPBN [88] contribute to dryness and orofacial pain following exposure to CIH.

## Conclusions

Peripheral increases in CGRP induced by CIH caused activation of SGCs and increased neuron–glia communication in the TG that affected all three branches of the trigeminal nerve and associated with sensitization of second-order neurons in laminae I–II of the Vc, which led to orofacial mechanical allodynia.

## Supplementary Material

zsad332_suppl_Supplementary_Materials

zsad332_suppl_Supplementary_Videos_1

zsad332_suppl_Supplementary_Videos_2

## Data Availability

The datasets generated and/or analyzed during the current study are available from the corresponding author on reasonable request.
