# Supplementary material for: Effect of chronic intermittent hypoxia on ocular and intraoral mechanical allodynia mediated via the calcitonin gene-related peptide in a rat
Source: Sleep. 2023 Dec 21;47(3):zsad332. doi: 10.1093/sleep/zsad332 (PMC10925949; doi:10.1093/sleep/zsad332)
Supplement: zsad332_suppl_Supplementary_Materials [file zsad332_suppl_supplementary_materials.pdf]

Effect of chronic intermittent hypoxia on ocular and intraoral mechanical allodynia mediated via the calcitonin gene-related peptide in a rat

Ayano Katagiri [1,*]; Saki Kishimoto [1,2]; Yoshie Okamoto [1]; Masaharu Yamada [1,2]; Hitoshi Niwa [2]; David A Bereiter [3]; Takafumi Kato [1]

1. Department of Oral Physiology, Osaka University Graduate School of Dentistry
2. Department of Dental Anesthesiology, Osaka University Graduate School of Dentistry
3. Department of Diagnostic and Biological Sciences, University of Minnesota School of Dentistry

Ayano Katagiri *: Corresponding author
katagiri.ayano.dent@osaka-u.ac.jp

List of Supplementary Material (Figures, Videos, illustration, and Tables)

- ✧ Supplementary Material Fig. S1: General conditions

- ✧ Supplementary illustration: high-resolution illustrations of Fig. 3C

- ✧ Supplementary Video 1: drinking water from a normal spout
- ✧ Supplementary Video 2: drinking water from a spout with mechanical stimulation (optical fibers)

- ✧ Supplementary Material Table S1: actual numbers and p-values in Fig. 1
- ✧ Supplementary Material Table S2-1: actual numbers and p-values in Fig. 2A-B
- ✧ Supplementary Material Table S2-2: actual numbers and p-values in Fig. 2C
- ✧ Supplementary Material Table S3: actual numbers and p-values in Fig. 3
- ✧ Supplementary Material Table S4: actual numbers and p-values in Fig. 4
- ✧ Supplementary Material Table S5-1: actual numbers and p-values in Fig. 5B
- ✧ Supplementary Material Table S5-2: actual numbers and p-values in Fig. 5C
- ✧ Supplementary Material Table S6: actual numbers and p-values in Fig. 6
- ✧ Supplementary Material Table S7: actual numbers and p-values in Fig. 7
- ✧ Supplementary Material Table S8: actual numbers and p-values in Fig. 8
- ✧ Supplementary Material Table S9: actual numbers and p-values in Supplementary Material Fig. S1

Supplementary Material Fig. S1

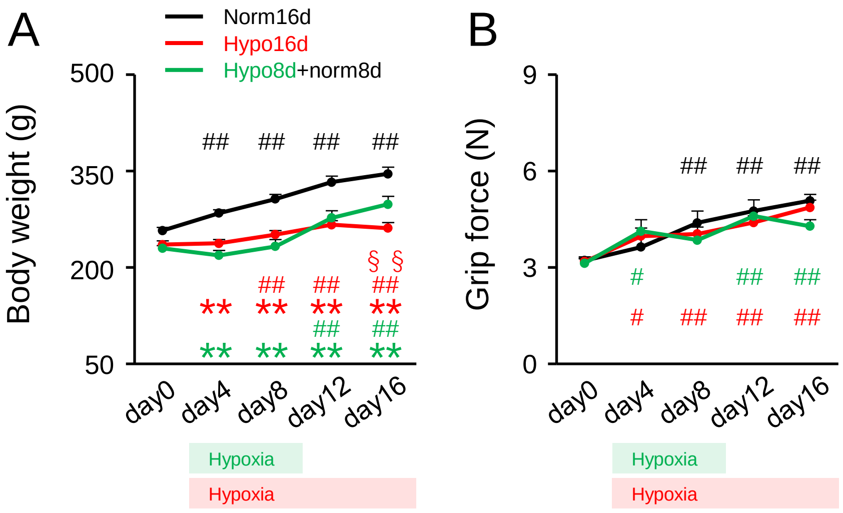

Supplementary Material Fig. S1: General conditions

A: Body weight. The red and green crossbars at the bottom of the graph indicate periods of intermittent hypoxia in the Hypo16d and Hypo8d+norm8d groups, respectively. Norm16d: n = 5, Hypo16d: n = 9, Hypo8d+norm8d: n = 6.

** $p < 0.01$: vs. Norm16d. ## $p < 0.01$: vs. day 0. §§ $p < 0.01$: vs. Hypo8d+norm8d.

B: Grip force.

# $p < 0.05$, ## $p < 0.01$: vs. day 0.

Norm16d: normoxia 16 days, Hypo16d: hypoxia 16 days, Hypo8+norm8: hypoxia 8 days and normoxia 8 days

Supplementary Material Table S1

Supplementary Material Table S1 (Fig. 1)

Fig. 1D SaO_2

	pre			post			# p-value (vs. pre)	
Normoxia-Normoxia	87.43	±	1.36	87.73	±	1.61	p >	0.9999
Normoxia-Hypoxia	82.84	±	4.33	40.55	±	9.01	p <	0.0001
* p-value (vs. Normoxia-Normoxia)	p >	0.9999		p <	0.0001			

Fig. 1D $PaCO_2$

	pre			post			# p-value (vs. pre)	
Normoxia-Normoxia	68.06	±	2.69	68.15	±	3.32	p >	0.9999
Normoxia-Hypoxia	61.34	±	1.51	43.00	±	1.94	p <	0.0001
* p-value (vs. Normoxia-Normoxia)	p =	0.1435		p <	0.0001			

Fig. 1D pH

	pre			post			# p-value (vs. pre)	
Normoxia-Normoxia	7.25	±	0.01	7.25	±	0.01	p >	0.9999
Normoxia-Hypoxia	7.28	±	0.01	7.35	±	0.03	p =	0.019
* p-value (vs. Normoxia-Normoxia)	p =	0.6062		p =	0.0014			

Fig. 1E Locomotor activity (pre)

	day -1 (14:00-20:00)			day -1/0 (20:00-02:00)			day 0 (02:00-08:00)			day 0 (08:00-14:00)		
Norm16d	520.90	±	41.59	4844.07	±	829.50	4651.17	±	562.26	523.25	±	114.67
Hypo16d	647.83	±	37.86	4342.03	±	514.90	4038.80	±	140.61	786.30	±	51.58
Hypo8d+norm8d	672.61	±	87.11	3794.31	±	964.95	3676.75	±	1021.40	886.79	±	218.84
p-value (Norm16d vs. Hypo16d)	p =	0.2525		p >	0.9999		p =	0.8637		p =	0.1887	
p-value (Norm16d vs. Hypo8d+norm8d)	p =	0.2486		p >	0.9999		p >	0.9999		p =	0.4549	
p-value (Hypo16d vs. Hypo8d+norm8d)	p >	0.9999		p >	0.9999		p >	0.9999		p >	0.9999	

Fig. 1F Locomotor activity (post)

	day 15 (14:00-20:00)			day 15/16 (20:00-02:00)			day 16 (02:00-08:00)			day 16 (08:00-14:00)		
Norm16d	455.83	±	93.78	4322	±	311.35	3597.63	±	374.15	644.1	±	159.26
Hypo16d	497.8	±	43.65	2083.17	±	132.92	2172.53	±	280.52	488.5	±	81.82
Hypo8d+norm8d	720.08	±	94.01	3719.19	±	334.83	3373.61	±	294.96	611.08	±	150.3
p-value (Norm16d vs. Hypo16d)	p >	0.9999		p =	0.0093		p =	0.0288		p =	0.7765	
p-value (Norm16d vs. Hypo8d+norm8d)	p =	0.0662		p =	0.8403		p >	0.9999		p >	0.9999	
p-value (Hypo16d vs. Hypo8d+norm8d)	p =	0.3317		p =	0.0923		p =	0.0682		p >	0.9999	

Supplementary Material Table S2-1

Supplementary Material Table S2-1 (Fig. 2)

Fig. 2A Tear volume

	tear volume
Norm16d	15.29 ± 0.44
Hypo8d	10.33 ± 0.87
Hypo16d	13.17 ± 0.36
Hypo8d+norm8d	14.30 ± 0.92
p-value (Norm16d vs. Hypo8d)	p = 0.0055
p-value (Norm16d vs. Hypo16d)	p = 0.3597
p-value (Norm16d vs. Hypo8d+norm8d)	p > 0.9999
p-value (Hypo8d vs. Hypo16d)	p = 0.914
p-value (Hypo8d vs. Hypo8d+norm8d)	p = 0.0532
p-value (Hypo16d vs. Hypo8d+norm8d)	p > >0.9999

Fig. 2B Eyeblink (hypertonic saline 0.15 M NaCl)

	day0	day4	# p-value (vs. day0)	day8	# p-value (vs. day0)	day12	# p-value (vs. day0)	day16	# p-value (vs. day0)
Norm16d	10.33 ± 1.52	11.50 ± 1.48	p > 0.9999	11.00 ± 1.15	p > 0.9999	9.83 ± 1.01	p > 0.9999	10.17 ± 1.14	p > 0.9999
Hypo16d	9.14 ± 0.80	12.00 ± 0.72	p = 0.3138	10.86 ± 0.74	p > 0.9999	10.71 ± 0.64	p > 0.9999	13.57 ± 2.10	p = 0.0292
Hypo8d+norm8d	9.00 ± 1.71	10.00 ± 1.15	p > 0.9999	10.50 ± 0.76	p > 0.9999	9.33 ± 0.84	p > 0.9999	12.17 ± 1.30	p = 0.2846
* p-value (Norm16d vs. Hypo16d)	p > 0.9999	p > 0.9999		p > 0.9999		p > 0.9999		p = 0.7070	
* p-value (Norm16d vs. Hypo8d+norm8d)	p > 0.9999	p > 0.9999		p > 0.9999		p > 0.9999		p > 0.9999	
§ p-value (Hypo16d vs. Hypo8d+norm8d)	p > 0.9999	p > 0.9999		p > 0.9999		p > 0.9999		p > 0.9999	

Fig. 2B Eyeblink (hypertonic saline 1.0 M NaCl)

	day0	day4	# p-value (vs. day0)	day8	# p-value (vs. day0)	day12	# p-value (vs. day0)	day16	# p-value (vs. day0)
Norm16d	26.17 ± 2.24	26.00 ± 1.55	p > 0.9999	26.00 ± 1.84	p > 0.9999	26.67 ± 2.59	p > 0.9999	27.00 ± 1.95	p > 0.9999
Hypo16d	23.14 ± 1.62	35.86 ± 3.03	p = 0.0009	39.86 ± 3.41	p < 0.0001	38.00 ± 3.60	p < 0.0001	31.14 ± 2.22	p = 0.0646
Hypo8d+norm8d	27.83 ± 2.02	36.33 ± 3.04	p = 0.0716	38.00 ± 3.44	p = 0.0200	31.17 ± 2.82	p > 0.9999	25.17 ± 0.83	p > 0.9999
* p-value (Norm16d vs. Hypo16d)	p > 0.9999	p = 0.1197		p = 0.0039		p = 0.0367		p > 0.9999	
* p-value (Norm16d vs. Hypo8d+norm8d)	p > 0.9999	p = 0.1108		p = 0.0303		p > 0.9999		p > 0.9999	
§ p-value (Hypo16d vs. Hypo8d+norm8d)	p > 0.9999	p > 0.9999		p > 0.9999		p = 0.9430		p > 0.9999	

Fig. 2B Eyeblink (hypertonic saline 2.5 M NaCl)

	day0	day4	# p-value (vs. day0)	day8	# p-value (vs. day0)	day12	# p-value (vs. day0)	day16	# p-value (vs. day0)
Norm16d	44.83 ± 1.70	42.00 ± 1.46	p > 0.9999	41.67 ± 1.93	p > 0.9999	42.50 ± 2.08	p > 0.9999	43.83 ± 1.40	p > 0.9999
Hypo16d	43.29 ± 1.69	54.14 ± 2.52	p = 0.0217	56.43 ± 3.10	p = 0.0036	60.43 ± 4.17	p = 0.0001	49.71 ± 3.45	p = 0.3730
Hypo8d+norm8d	46.83 ± 1.56	55.50 ± 4.39	p = 0.1492	54.83 ± 2.94	p = 0.2159	49.83 ± 3.66	p > 0.9999	41.50 ± 1.45	p = 0.7811
* p-value (Norm16d vs. Hypo16d)	p > 0.9999	p = 0.0347		p = 0.0039		p = 0.0002		p > 0.9999	
* p-value (Norm16d vs. Hypo8d+norm8d)	p > 0.9999	p = 0.0173		p = 0.0225		p > 0.9999		p > 0.9999	
§ p-value (Hypo16d vs. Hypo8d+norm8d)	p > 0.9999	p > 0.9999		p > 0.9999		p = 0.1117		p = 0.5448	

Supplementary Material Table S2-2

Supplementary Material Table S2-2 **(Fig. 2)**

Fig. 2C Eyeblink (mechanical stimulation 0.008 g)

	day0	day4	# p-value (vs. day0)	day8	# p-value (vs. day0)	day12	# p-value (vs. day0)	day16	# p-value (vs. day0)
Norm16d	1.07 ± 0.11	0.93 ± 0.08	p > 0.9999	0.73 ± 0.07	p = 0.1437	1.00 ± 0.07	p > 0.9999	0.97 ± 0.12	p > 0.9999
Hypo16d	1.00 ± 0.15	1.49 ± 0.23	p = 0.0051	1.29 ± 0.13	p = 0.2062	1.14 ± 0.11	p > 0.9999	1.11 ± 0.06	p > 0.9999
Hypo8d+norm8d	0.93 ± 0.07	1.33 ± 0.10	p = 0.0498	1.43 ± 0.10	p = 0.0082	1.13 ± 0.10	p = 0.8126	0.93 ± 0.08	p > 0.9999
* p-value (Norm16d vs. Hypo16d)	p > 0.9999	p = 0.0208		p = 0.0208		p > 0.9999		p > 0.9999	
* p-value (Norm16d vs. Hypo8d+norm8d)	p > 0.9999	p = 0.3501		p = 0.0018		p > 0.9999		p > 0.9999	
§ p-value (Hypo16d vs. Hypo8d+norm8d)	p > 0.9999	p > 0.9999		p > 0.9999		p > 0.9999		p > 0.9999	

Fig. 2C Eyeblink (mechanical stimulation 0.02 g)

	day0	day4	# p-value (vs. day0)	day8	# p-value (vs. day0)	day12	# p-value (vs. day0)	day16	# p-value (vs. day0)
Norm16d	1.77 ± 0.06	1.40 ± 0.04	p = 0.1473	1.33 ± 0.21	p = 0.057	1.57 ± 0.08	p = 0.9965	1.47 ± 0.08	p = 0.3435
Hypo16d	1.26 ± 0.10	2.11 ± 0.18	p < 0.0001	2.17 ± 0.18	p < 0.0001	2.00 ± 0.20	p < 0.0001	1.69 ± 0.12	p = 0.0362
Hypo8d+norm8d	1.53 ± 0.04	2.20 ± 0.09	p = 0.0010	2.37 ± 0.11	p < 0.0001	1.57 ± 0.06	p > 0.9999	1.83 ± 0.14	p = 0.3435
* p-value (Norm16d vs. Hypo16d)	p = 0.0986	p = 0.0029		p = 0.0002		p = 0.3003		p > 0.9999	
* p-value (Norm16d vs. Hypo8d+norm8d)	p > 0.9999	p = 0.0010		p < 0.0001		p > 0.9999		p = 0.84732	
§ p-value (Hypo16d vs. Hypo8d+norm8d)	p > 0.9999	p > 0.9999		p > 0.9999		p = 0.3003		p > 0.9999	

Fig. 2C Eyeblink (mechanical stimulation 0.04 g)

	day0	day4	# p-value (vs. day0)	day8	# p-value (vs. day0)	day12	# p-value (vs. day0)	day16	# p-value (vs. day0)
Norm16d	2.20 ± 0.05	2.05 ± 0.07	p > 0.9999	1.93 ± 0.18	p = 0.5755	2.17 ± 0.08	p > 0.9999	2.40 ± 0.14	p > 0.9999
Hypo16d	2.03 ± 0.12	3.09 ± 0.31	p < 0.0001	3.23 ± 0.29	p < 0.0001	2.94 ± 0.20	p < 0.0001	2.51 ± 0.19	p = 0.0198
Hypo8d+norm8d	2.10 ± 0.07	3.03 ± 0.10	p < 0.0001	2.87 ± 0.11	p = 0.0003	2.07 ± 0.07	p > 0.9999	2.27 ± 0.08	p > 0.9999
* p-value (Norm16d vs. Hypo16d)	p > 0.9999	p = 0.0005		p < 0.0001		p = 0.0221		p > 0.9999	
* p-value (Norm16d vs. Hypo8d+norm8d)	p > 0.9999	p = 0.002		p = 0.004		p > 0.9999		p > 0.9999	
§ p-value (Hypo16d vs. Hypo8d+norm8d)	p > 0.9999	p > 0.9999		p > 0.9999		p = 0.0056		p > 0.9999	

Supplementary Material Table S3

Supplementary Material Table S3 **(Fig. 3)**

Fig. 3A Saliva volume

	Saliva volume
Norm16d	12.17 ± 0.87
Hypo8d	13.00 ± 1.58
Hypo16d	6.00 ± 0.89
Hypo8d+norm8d	5.71 ± 0.71
p-value (Norm16d vs. Hypo8d)	p > 0.9999
p-value (Norm16d vs. Hypo16d)	p = 0.0404
p-value (Norm16d vs. Hypo8d+norm8d)	p = 0.0151
p-value (Hypo8d vs. Hypo16d)	p = 0.0416
p-value (Hypo8d vs. Hypo8d+norm8d)	p = 0.0170
p-value (Hypo16d vs. Hypo8d+norm8d)	p > 0.9999

Fig. 3B % of water intake (mechanical stimulation)

	day-1&0	day 3&4	# p-value (vs. day0)	day7&8	# p-value (vs. day0)	day 11&12	# p-value (vs. day0)	day 15&16	# p-value (vs. day0)
Norm16d	79.69 ± 3.02	85.32 ± 2.93	p = 0.9805	82.27 ± 4.11	p > 0.9999	82.01 ± 3.19	p > 0.9999	80.39 ± 2.75	p > 0.9999
Hypo16d	80.23 ± 1.83	47.74 ± 6.60	p < 0.0001	42.55 ± 9.35	p < 0.0001	36.66 ± 7.23	p < 0.0001	36.44 ± 6.60	p < 0.0001
Hypo8d+norm8d	77.81 ± 2.13	40.25 ± 11.03	p < 0.0001	33.29 ± 6.58	p < 0.0001	35.05 ± 7.66	p < 0.0001	32.82 ± 8.54	p < 0.0001
* p-value (Norm16d vs. Hypo16d)	p > 0.9999	p = 0.0001		p < 0.0001		p < 0.0001		p < 0.0001	
* p-value (Norm16d vs. Hypo8d+norm8d)	p > 0.9999	p < 0.0001		p < 0.0001		p < 0.0001		p < 0.0001	
§ p-value (Hypo16d vs. Hypo8d+norm8d)	p > 0.9999	p > 0.9999		p > 0.9999		p > 0.9999		p > 0.9999	

Supplementary Material Table S4

Supplementary Material Table S4 **(Fig. 4)**

Fig. 4B TG GFAP (ophthalmic nerve)

	%		
Norm16d	2.47	±	0.53
Hypo8d	42.76	±	12.97
Hypo16d	50.31	±	10.95
Hypo8d+norm8d	3.06	±	0.98
p-value (Norm16d vs. Hypo8d)	p =	0.0351	
p-value (Norm16d vs. Hypo16d)	p =	0.0093	
p-value (Norm16d vs. Hypo8d+norm8d)	p >	0.9999	
p-value (Hypo8d vs. Hypo16d)	p >	0.9999	
p-value (Hypo8d vs. Hypo8d+norm8d)	p =	0.0397	
p-value (Hypo16d vs. Hypo8d+norm8d)	p =	0.0107	

Fig. 4B TG GFAP (maxillary nerve)

	%		
Norm16d	3.43	±	2.42
Hypo8d	38.23	±	13.36
Hypo16d	44.15	±	7.49
Hypo8d+norm8d	3.20	±	1.01
p-value (Norm16d vs. Hypo8d)	p =	0.0291	
p-value (Norm16d vs. Hypo16d)	p =	0.0076	
p-value (Norm16d vs. Hypo8d+norm8d)	p >	0.9999	
p-value (Hypo8d vs. Hypo16d)	p >	0.9999	
p-value (Hypo8d vs. Hypo8d+norm8d)	p =	0.1198	
p-value (Hypo16d vs. Hypo8d+norm8d)	p =	0.0374	

Fig. 4B TG GFAP (mandibular nerve)

	%		
Norm16d	1.58	±	0.68
Hypo8d	35.13	±	7.95
Hypo16d	34.02	±	8.68
Hypo8d+norm8d	1.54	±	0.40
p-value (Norm16d vs. Hypo8d)	p =	0.0115	
p-value (Norm16d vs. Hypo16d)	p =	0.0151	
p-value (Norm16d vs. Hypo8d+norm8d)	p >	0.9999	
p-value (Hypo8d vs. Hypo16d)	p >	0.9999	
p-value (Hypo8d vs. Hypo8d+norm8d)	p =	0.0256	
p-value (Hypo16d vs. Hypo8d+norm8d)	p =	0.0330	

Fig. 4D TG CGRP (ophthalmic nerve)

	%		
Norm16d	16.83	±	1.15
Hypo8d	48.75	±	1.50
Hypo16d	50.11	±	4.23
Hypo8d+norm8d	47.88	±	2.85
p-value (Norm16d vs. Hypo8d)	p =	0.0330	
p-value (Norm16d vs. Hypo16d)	p =	0.0042	
p-value (Norm16d vs. Hypo8d+norm8d)	p =	0.0478	
p-value (Hypo8d vs. Hypo16d)	p >	0.9999	
p-value (Hypo8d vs. Hypo8d+norm8d)	p >	0.9999	
p-value (Hypo16d vs. Hypo8d+norm8d)	p >	0.9999	

Fig. 4D TG CGRP (maxillary nerve)

	%		
Norm16d	16.44	±	1.47
Hypo8d	46.48	±	3.79
Hypo16d	47.83	±	3.73
Hypo8d+norm8d	45.97	±	1.12
p-value (Norm16d vs. Hypo8d)	p =	0.0132	
p-value (Norm16d vs. Hypo16d)	p =	0.0151	
p-value (Norm16d vs. Hypo8d+norm8d)	p =	0.0374	
p-value (Hypo8d vs. Hypo16d)	p >	0.9999	
p-value (Hypo8d vs. Hypo8d+norm8d)	p >	0.9999	
p-value (Hypo16d vs. Hypo8d+norm8d)	p >	0.9999	

Fig. 4D TG CGRP (mandibular nerve)

	%		
Norm16d	18.86	±	2.08
Hypo8d	51.70	±	4.10
Hypo16d	53.07	±	3.76
Hypo8d+norm8d	52.63	±	2.53
p-value (Norm16d vs. Hypo8d)	p =	0.0374	
p-value (Norm16d vs. Hypo16d)	p =	0.0132	
p-value (Norm16d vs. Hypo8d+norm8d)	p =	0.0151	
p-value (Hypo8d vs. Hypo16d)	p >	0.9999	
p-value (Hypo8d vs. Hypo8d+norm8d)	p >	0.9999	
p-value (Hypo16d vs. Hypo8d+norm8d)	p >	0.9999	

Fig. 4E TG CGRP cell size (ophthalmic nerve)

	< 400 μm2	400 - 800 μm2	> 800 μm2
Norm16d	15.74%	44.14%	40.12%
Hypo8d	19.83%	46.08%	34.09%
p-value	1.000		

Fig. 4E TG CGRP cell size (maxillary nerve)

	< 400 μm2	400 - 800 μm2	> 800 μm2
Norm16d	17.03%	53.00%	29.97%
Hypo8d	24.02%	49.89%	26.09%
p-value	1.000		

Fig. 4E TG CGRP cell size (mandibular nerve)

	< 400 μm2	400 - 800 μm2	> 800 μm2
Norm16d	19.35%	52.49%	28.15%
Hypo8d	27.78%	52.63%	19.59%
p-value	1.000		

Supplementary Material Table S5-1

Supplementary Material Table S5-1 **(Fig. 5B)**

Fig. 5B Vc cFos distribution (left+right, surface+deep)

	2.0			1.5			1.0			0.5			0 (obex)			P0.5			P1.0			P1.5			P2.0		
Norm16d	5.19	±	1.32	5.25	±	1.03	7.42	±	2.70	11.58	±	2.19	24.08	±	6.86	34.00	±	6.99	42.08	±	5.14	37.25	±	3.15	29.42	±	5.40
Hypo8d	3.36	±	1.65	2.07	±	0.79	4.64	±	1.48	15.93	±	3.46	35.74	±	7.02	45.29	±	3.04	42.57	±	7.93	37.79	±	4.22	36.00	±	3.97
Hypo16d	6.25	±	2.56	6.33	±	3.12	7.17	±	2.21	20.86	±	4.53	45.72	±	5.85	78.11	±	9.35	55.00	±	6.78	62.92	±	10.52	51.08	±	12.66
Hypo8d+norm8d	1.00	±	0.62	1.08	±	0.42	1.08	±	0.35	6.17	±	2.71	19.75	±	6.28	42.25	±	10.07	37.33	±	12.09	35.58	±	11.46	25.25	±	8.52

	P2.5			P3.0			P3.5			P4.0			P4.5			P5.0			P5.5			P6.0			P6.5		
Norm16d	30.08	±	3.97	26.75	±	2.42	28.11	±	2.73	22.96	±	4.15	22.86	±	0.99	27.31	±	4.16	19.22	±	4.00	23.08	±	4.77	21.08	±	3.88
Hypo8d	38.64	±	4.92	38.74	±	5.43	34.24	±	6.13	34.26	±	6.81	26.50	±	4.52	20.14	±	3.77	15.43	±	3.22	15.60	±	3.25	12.90	±	2.77
Hypo16d	36.25	±	10.09	45.50	±	11.42	44.50	±	11.51	41.14	±	8.35	47.31	±	8.78	40.00	±	7.01	31.21	±	6.21	30.25	±	7.90	26.75	±	5.40
Hypo8d+norm8d	34.28	±	8.96	24.08	±	8.49	25.50	±	8.11	22.39	±	3.10	19.74	±	2.27	15.38	±	2.62	12.36	±	3.42	11.75	±	2.70	10.42	±	2.42

Supplementary Material Table S5-2

Supplementary Material Table S5-2 (Fig. 5C)

Fig. 5C Vc cFos (Vi)

	Mean number		
Norm16d	5.95	±	0.93
Hypo8d	3.36	±	1.23
Hypo16d	6.58	±	2.53
Hypo8d+norm8d	1.06	±	0.37
p-value (Norm16d vs. Hypo8d)	p =	0.9647	
p-value (Norm16d vs. Hypo16d)	p >	0.9999	
p-value (Norm16d vs. Hypo8d+norm8d)	p =	0.0182	
p-value (Hypo8d vs. Hypo16d)	p >	0.9999	
p-value (Hypo8d vs. Hypo8d+norm8d)	p =	0.5659	
p-value (Hypo16d vs. Hypo8d+norm8d)	p =	0.0897	

Fig. 5C Vc cFos (Vi/Vc)

	Mean number		
Norm16d	23.22	±	4.02
Hypo8d	32.32	±	2.03
Hypo16d	48.23	±	5.99
Hypo8d+norm8d	22.72	±	5.32
p-value (Norm16d vs. Hypo8d)	p =	0.9357	
p-value (Norm16d vs. Hypo16d)	p =	0.0196	
p-value (Norm16d vs. Hypo8d+norm8d)	p >	0.9999	
p-value (Hypo8d vs. Hypo16d)	p =	0.6136	
p-value (Hypo8d vs. Hypo8d+norm8d)	p >	0.9999	
p-value (Hypo16d vs. Hypo8d+norm8d)	p =	0.0222	

Fig. 5C Vc cFos (Mid-Vc laminae I-II)

	Mean number		
Norm16d	10.98	±	1.10
Hypo8d	14.62	±	1.67
Hypo16d	24.61	±	2.72
Hypo8d+norm8d	13.52	±	3.16
p-value (Norm16d vs. Hypo8d)	p >	0.9999	
p-value (Norm16d vs. Hypo16d)	p =	0.0152	
p-value (Norm16d vs. Hypo8d+norm8d)	p >	0.9999	
p-value (Hypo8d vs. Hypo16d)	p =	0.2373	
p-value (Hypo8d vs. Hypo8d+norm8d)	p >	0.9999	
p-value (Hypo16d vs. Hypo8d+norm8d)	p =	0.0724	

Fig. 5C Vc cFos (Caud-Vc/C2 laminae I-II)

	Mean number		
Norm16d	11.78	±	1.11
Hypo8d	9.2	±	1.13
Hypo16d	22.41	±	3.93
Hypo8d+norm8d	8.76	±	1.15
p-value (Norm16d vs. Hypo8d)	p >	0.9999	
p-value (Norm16d vs. Hypo16d)	p =	0.2854	
p-value (Norm16d vs. Hypo8d+norm8d)	p >	0.9999	
p-value (Hypo8d vs. Hypo16d)	p =	0.0158	
p-value (Hypo8d vs. Hypo8d+norm8d)	p >	0.9999	
p-value (Hypo16d vs. Hypo8d+norm8d)	p =	0.0102	

Fig. 5C Vc cFos (Mid-Vc laminae III-V)

	Mean number		
Norm16d	19.97	±	2.25
Hypo8d	22.85	±	4.25
Hypo16d	23.45	±	6.33
Hypo8d+norm8d	15.69	±	5.52
p-value (Norm16d vs. Hypo8d)	p >	0.9999	
p-value (Norm16d vs. Hypo16d)	p >	0.9999	
p-value (Norm16d vs. Hypo8d+norm8d)	p >	0.9999	
p-value (Hypo8d vs. Hypo16d)	p >	0.9999	
p-value (Hypo8d vs. Hypo8d+norm8d)	p >	0.9999	
p-value (Hypo16d vs. Hypo8d+norm8d)	p >	0.9999	

Fig. 5C Vc cFos (Caud-Vc/C2 laminae III-V)

	Mean number		
Norm16d	10.93	±	2.02
Hypo8d	8.91	±	1.98
Hypo16d	12.7	±	3.07
Hypo8d+norm8d	5.17	±	1.23
p-value (Norm16d vs. Hypo8d)	p >	0.9999	
p-value (Norm16d vs. Hypo16d)	p >	0.9999	
p-value (Norm16d vs. Hypo8d+norm8d)	p =	0.2481	
p-value (Hypo8d vs. Hypo16d)	p >	0.9999	
p-value (Hypo8d vs. Hypo8d+norm8d)	p >	0.9999	
p-value (Hypo16d vs. Hypo8d+norm8d)	p =	0.1304	

Supplementary Material Table S6

Supplementary Material Table S6 (Fig. 6)

Fig. 6A Eyeblink (mechanical stimulation 0.008 g)

	Baseline	Post-cannulation	# p-value (vs. baseline)	day8	# p-value (vs. baseline)	day16	# p-value (vs. baseline)
CGRP8-37 Norm16d	0.81 ± 0.12	0.89 ± 0.16	p > >0.9999	0.72 ± 0.16	p = >0.9999	0.78 ± 0.07	p > >0.9999
CGRP8-37 Hypo16d	0.89 ± 0.11	0.78 ± 0.07	p = >0.9999	0.79 ± 0.08	p = >0.9999	1.06 ± 0.26	p > >0.9999
Saline Hypo16d	0.81 ± 0.10	0.74 ± 0.05	p = >0.9999	1.33 ± 0.15	p = 0.0211	1.02 ± 0.15	p = >0.9999
* p-value (CGRP8-37 Norm16d vs. CGRP8-37 Hypo16d)	p > 0.9999	p > 0.9999		p > 0.9999		p > 0.9999	
* p-value (CGRP8-37 Norm16d vs. Saline Hypo16d)	p > 0.9999	p > 0.9999		p = 0.0213		p > 0.9999	
& p-value (CGRP8-37 Hypo16d vs. Saline Hypo16d)	p > 0.9999	p > 0.9999		p = 0.0630		p > 0.9999	

Fig. 6A Eyeblink (mechanical stimulation 0.02 g)

	Baseline	Post-cannulation	# p-value (vs. baseline)	day8	# p-value (vs. baseline)	day16	# p-value (vs. baseline)
CGRP8-37 Norm16d	1.17 ± 0.19	1.22 ± 0.11	p = >0.9999	1.67 ± 0.17	p = 0.0248	1.38 ± 0.06	p = >0.9999
CGRP8-37 Hypo16d	1.56 ± 0.07	1.50 ± 0.07	p < >0.9999	1.67 ± 0.08	p < >0.9999	1.72 ± 0.16	p < >0.9999
Saline Hypo16d	1.38 ± 0.13	1.38 ± 0.09	p = >0.9999	2.37 ± 0.19	p < <0.0001	1.62 ± 0.11	p > 0.7553
* p-value (CGRP8-37 Norm16d vs. CGRP8-37 Hypo16d)	p = 0.5029	p > 0.9999		p > 0.9999		p = 0.8352	
* p-value (CGRP8-37 Norm16d vs. Saline Hypo16d)	p > 0.9999	p > 0.9999		p = 0.0029		p > 0.9999	
& p-value (CGRP8-37 Hypo16d vs. Saline Hypo16d)	p > 0.9999	p > 0.9999		p = 0.0030		p > 0.9999	

Fig. 6A Eyeblink (mechanical stimulation 0.04 g)

	Baseline	Post-cannulation	# p-value (vs. baseline)	day8	# p-value (vs. baseline)	day16	# p-value (vs. baseline)
CGRP8-37 Norm16d	2.00 ± 0.12	2.25 ± 0.22	p > >0.9999	2.54 ± 0.14	p = 0.146	2.22 ± 0.21	p > >0.9999
CGRP8-37 Hypo16d	2.17 ± 0.14	2.28 ± 0.16	p < >0.9999	2.33 ± 0.18	p < >0.9999	2.39 ± 0.22	p < >0.9999
Saline Hypo16d	2.10 ± 0.14	2.19 ± 0.10	p < >0.9999	3.18 ± 0.24	p = <0.0001	2.50 ± 0.16	p > 0.4072
* p-value (CGRP8-37 Norm16d vs. CGRP8-37 Hypo16d)	p > 0.9999	p > 0.9999		p > 0.9999		p > 0.9999	
* p-value (CGRP8-37 Norm16d vs. Saline Hypo16d)	p > 0.9999	p > 0.9999		p = 0.1374		p > 0.9999	
& p-value (CGRP8-37 Hypo16d vs. Saline Hypo16d)	p > 0.9999	p > 0.9999		p = 0.0118		p > 0.9999	

Fig. 6B % of water intake (mechanical stimulation)

	Baseline	Post-cannulation	# p-value (vs. baseline)	day7&8	# p-value (vs. baseline)	day 15&16	# p-value (vs. baseline)
CGRP8-37 Norm16d	73.60 ± 5.58	66.61 ± 4.39	p > 0.9999	65.39 ± 3.22	p > 0.9999	73.50 ± 4.06	p > 0.9999
CGRP8-37 Hypo16d	75.43 ± 3.89	72.60 ± 4.48	p > 0.9999	81.17 ± 7.19	p > 0.9999	84.12 ± 5.39	p > 0.9999
Saline Hypo16d	73.07 ± 4.52	71.04 ± 6.10	p > 0.9999	24.76 ± 7.54	p < 0.0001	30.58 ± 1.74	p < 0.0001
* p-value (CGRP8-37 Norm16d vs. CGRP8-37 Hypo16d)	p > 0.9999	p > 0.9999		p = 0.521		p > 0.9999	
* p-value (CGRP8-37 Norm16d vs. Saline Hypo16d)	p > 0.9999	p > 0.9999		p < 0.0001		p < 0.0001	
& p-value (CGRP8-37 Hypo16d vs. Saline Hypo16d)	p > 0.9999	p > 0.9999		p < 0.0001		p < 0.0001	

Supplementary Material Table S7

Supplementary Material Table S7 **(Fig. 7)**

Fig. 7A TG CGRP (ophthalmic nerve)

	%		
CGRP8-37 Norm16d	23.17	±	1.61
CGRP8-37 Hypo8d	24.68	±	1.95
CGRP8-37 Hypo16d	20.96	±	1.73
Saline Hypo16d	54.87	±	3.26
p-value (CGRP8-37 Norm16d vs. CGRP8-37 Hypo8d)	p >	0.9999	
p-value (CGRP8-37 Norm16d vs. CGRP8-37 Hypo16d)	p >	0.9999	
p-value (CGRP8-37 Norm16d vs. Saline Hypo16d)	p =	0.0177	
p-value (CGRP8-37 Hypo8d vs. CGRP8-37 Hypo16d)	p >	0.9999	
p-value (CGRP8-37 Hypo8d vs. Saline Hypo16d)	p =	0.0298	
p-value (CGRP8-37 Hypo16d vs. Saline Hypo16d)	p =	0.0044	

Fig. 7A TG CGRP (maxillary nerve)

	%		
CGRP8-37 Norm16d	23.25	±	2.12
CGRP8-37 Hypo8d	20.34	±	1.26
CGRP8-37 Hypo16d	22.51	±	1.76
Saline Hypo16d	54.11	±	2.81
p-value (CGRP8-37 Norm16d vs. CGRP8-37 Hypo8d)	p >	0.9999	
p-value (CGRP8-37 Norm16d vs. CGRP8-37 Hypo16d)	p >	0.9999	
p-value (CGRP8-37 Norm16d vs. Saline Hypo16d)	p =	0.0433	
p-value (CGRP8-37 Hypo8d vs. CGRP8-37 Hypo16d)	p >	0.9999	
p-value (CGRP8-37 Hypo8d vs. Saline Hypo16d)	p =	0.0028	
p-value (CGRP8-37 Hypo16d vs. Saline Hypo16d)	p =	0.0178	

Fig. 7A TG CGRP (mandibular nerve)

	%		
CGRP8-37 Norm16d	21.38	±	2.89
CGRP8-37 Hypo8d	20.67	±	2.08
CGRP8-37 Hypo16d	21.35	±	1.28
Saline Hypo16d	61.59	±	2.37
p-value (CGRP8-37 Norm16d vs. CGRP8-37 Hypo8d)	p >	0.9999	
p-value (CGRP8-37 Norm16d vs. CGRP8-37 Hypo16d)	p >	0.9999	
p-value (CGRP8-37 Norm16d vs. Saline Hypo16d)	p =	0.0038	
p-value (CGRP8-37 Hypo8d vs. CGRP8-37 Hypo16d)	p >	0.9999	
p-value (CGRP8-37 Hypo8d vs. Saline Hypo16d)	p =	0.0119	
p-value (CGRP8-37 Hypo16d vs. Saline Hypo16d)	p =	0.0489	

Fig. 7B TG CGRP cell size (ophthalmic nerve)

	< 400 μm2	400 - 800 μm2	> 800 μm2
CGRP8-37 Norm16d	34.8%	42.9%	22.3%
CGRP8-37 Hypo8d	34.8%	42.9%	22.3%
CGRP8-37 Hypo16d	34.8%	42.9%	22.3%
Saline Hypo16d	34.8%	42.9%	22.3%
p-value	1.000		

Fig. 7B TG CGRP cell size (maxillary nerve)

	< 400 μm2	400 - 800 μm2	> 800 μm2
CGRP8-37 Norm16d	34.7%	44.2%	21.1%
CGRP8-37 Hypo8d	34.7%	44.2%	21.1%
CGRP8-37 Hypo16d	34.7%	44.2%	21.1%
Saline Hypo16d	34.7%	44.2%	21.1%
p-value	1.000		

Fig. 7B TG CGRP cell size (mandibular nerve)

	< 400 μm2	400 - 800 μm2	> 800 μm2
CGRP8-37 Norm16d	29.0%	49.7%	21.3%
CGRP8-37 Hypo8d	29.0%	49.7%	21.3%
CGRP8-37 Hypo16d	29.0%	49.7%	21.3%
Saline Hypo16d	29.0%	49.7%	21.3%
p-value	1.000		

Fig. 7C TG GFAP (ophthalmic nerve)

	%		
CGRP8-37 Norm16d	3.72	±	1.13
CGRP8-37 Hypo8d	3.27	±	0.44
CGRP8-37 Hypo16d	8.90	±	3.10
Saline Hypo16d	47.61	±	8.62
p-value (CGRP8-37 Norm16d vs. CGRP8-37 Hypo8d)	p >	0.9999	
p-value (CGRP8-37 Norm16d vs. CGRP8-37 Hypo16d)	p >	0.9999	
p-value (CGRP8-37 Norm16d vs. Saline Hypo16d)	p =	0.0057	
p-value (CGRP8-37 Hypo8d vs. CGRP8-37 Hypo16d)	p >	0.9999	
p-value (CGRP8-37 Hypo8d vs. Saline Hypo16d)	p =	0.0023	
p-value (CGRP8-37 Hypo16d vs. Saline Hypo16d)	p =	0.1826	

Fig. 7C TG GFAP (maxillary nerve)

	%		
CGRP8-37 Norm16d	5.10	±	1.60
CGRP8-37 Hypo8d	4.18	±	0.76
CGRP8-37 Hypo16d	11.53	±	3.89
Saline Hypo16d	46.10	±	3.81
p-value (CGRP8-37 Norm16d vs. CGRP8-37 Hypo8d)	p >	0.9999	
p-value (CGRP8-37 Norm16d vs. CGRP8-37 Hypo16d)	p >	0.9999	
p-value (CGRP8-37 Norm16d vs. Saline Hypo16d)	p =	0.003	
p-value (CGRP8-37 Hypo8d vs. CGRP8-37 Hypo16d)	p >	0.9999	
p-value (CGRP8-37 Hypo8d vs. Saline Hypo16d)	p =	0.0051	
p-value (CGRP8-37 Hypo16d vs. Saline Hypo16d)	p =	0.1154	

Fig. 7C TG GFAP (mandibular nerve)

	%		
CGRP8-37 Norm16d	4.68	±	1.86
CGRP8-37 Hypo8d	3.28	±	0.75
CGRP8-37 Hypo16d	13.15	±	5.43
Saline Hypo16d	45.36	±	4.27
p-value (CGRP8-37 Norm16d vs. CGRP8-37 Hypo8d)	p >	0.9999	
p-value (CGRP8-37 Norm16d vs. CGRP8-37 Hypo16d)	p >	0.9999	
p-value (CGRP8-37 Norm16d vs. Saline Hypo16d)	p =	0.0045	
p-value (CGRP8-37 Hypo8d vs. CGRP8-37 Hypo16d)	p =	0.9111	
p-value (CGRP8-37 Hypo8d vs. Saline Hypo16d)	p =	0.0025	
p-value (CGRP8-37 Hypo16d vs. Saline Hypo16d)	p =	0.2446	

Supplementary Material Table S8

Supplementary Material Table S8 **(Fig. 8)**

Fig. 8B Vc CGRP-positive fiber (Posterior -1.0 from obex)

	mean occypancy		
CGRP8-37 Norm16d	34.43	±	5.24
CGRP8-37 Hypo8d	20.21	±	3.49
CGRP8-37 Hypo16d	15.53	±	1.61
Saline Hypo16d	48.24	±	3.06
p-value (CGRP8-37 Norm16d vs. CGRP8-37 Hypo8d)	p =	0.4402	
p-value (CGRP8-37 Norm16d vs. CGRP8-37 Hypo16d)	p =	0.0684	
p-value (CGRP8-37 Norm16d vs. Saline Hypo16d)	p >	0.9999	
p-value (CGRP8-37 Hypo8d vs. CGRP8-37 Hypo16d)	p >	0.9999	
p-value (CGRP8-37 Hypo8d vs. Saline Hypo16d)	p =	0.0204	
p-value (CGRP8-37 Hypo16d vs. Saline Hypo16d)	p =	0.0011	

Fig. 8B Vc CGRP-positive fiber CGRP (Posterior -3.0 from obex)

	mean occypancy		
CGRP8-37 Norm16d	21.36	±	3.53
CGRP8-37 Hypo8d	22.10	±	3.00
CGRP8-37 Hypo16d	15.98	±	1.95
Saline Hypo16d	42.16	±	2.03
p-value (CGRP8-37 Norm16d vs. CGRP8-37 Hypo8d)	p >	0.9999	
p-value (CGRP8-37 Norm16d vs. CGRP8-37 Hypo16d)	p >	0.9999	
p-value (CGRP8-37 Norm16d vs. Saline Hypo16d)	p =	0.0354	
p-value (CGRP8-37 Hypo8d vs. CGRP8-37 Hypo16d)	p >	0.9999	
p-value (CGRP8-37 Hypo8d vs. Saline Hypo16d)	p =	0.0661	
p-value (CGRP8-37 Hypo16d vs. Saline Hypo16d)	p =	0.0014	

Fig. 8B Vc CGRP-positive fiber CGRP (Posterior -5.0 from obex)

	mean occypancy		
CGRP8-37 Norm16d	27.98	±	3.32
CGRP8-37 Hypo8d	27.12	±	4.72
CGRP8-37 Hypo16d	25.69	±	4.12
Saline Hypo16d	54.25	±	4.19
p-value (CGRP8-37 Norm16d vs. CGRP8-37 Hypo8d)	p >	0.9999	
p-value (CGRP8-37 Norm16d vs. CGRP8-37 Hypo16d)	p >	0.9999	
p-value (CGRP8-37 Norm16d vs. Saline Hypo16d)	p =	0.0225	
p-value (CGRP8-37 Hypo8d vs. CGRP8-37 Hypo16d)	p >	0.9999	
p-value (CGRP8-37 Hypo8d vs. Saline Hypo16d)	p =	0.0272	
p-value (CGRP8-37 Hypo16d vs. Saline Hypo16d)	p =	0.0066	

Fig. 8C Vc cFos (Vi)

	mean number		
CGRP8-37 Norm16d	0.15	±	0.09
CGRP8-37 Hypo8d	1.16	±	0.56
CGRP8-37 Hypo16d	6.44	±	1.47
Saline Hypo16d	8.03	±	1.55
p-value (CGRP8-37 Norm16d vs. CGRP8-37 Hypo8d)	p >	0.9999	
p-value (CGRP8-37 Norm16d vs. CGRP8-37 Hypo16d)	p =	0.0097	
p-value (CGRP8-37 Norm16d vs. Saline Hypo16d)	p =	0.0014	
p-value (CGRP8-37 Hypo8d vs. CGRP8-37 Hypo16d)	p =	0.2442	
p-value (CGRP8-37 Hypo8d vs. Saline Hypo16d)	p =	0.0595	
p-value (CGRP8-37 Hypo16d vs. Saline Hypo16d)	p >	0.9999	

Fig. 8C Vc cFos (Vi/Vc)

	mean number		
CGRP8-37 Norm16d	1.17	±	0.08
CGRP8-37 Hypo8d	9.33	±	1.84
CGRP8-37 Hypo16d	38.34	±	3.36
Saline Hypo16d	62.33	±	7.21
p-value (CGRP8-37 Norm16d vs. CGRP8-37 Hypo8d)	p =	0.8493	
p-value (CGRP8-37 Norm16d vs. CGRP8-37 Hypo16d)	p =	0.0151	
p-value (CGRP8-37 Norm16d vs. Saline Hypo16d)	p <	0.0001	
p-value (CGRP8-37 Hypo8d vs. CGRP8-37 Hypo16d)	p =	0.7244	
p-value (CGRP8-37 Hypo8d vs. Saline Hypo16d)	p =	0.0256	
p-value (CGRP8-37 Hypo16d vs. Saline Hypo16d)	p >	0.9999	

Fig. 8C Vc cFos (Mid-Vc laminae I-II)

	mean number		
CGRP8-37 Norm16d	0.98	±	0.20
CGRP8-37 Hypo8d	7.62	±	1.16
CGRP8-37 Hypo16d	17.39	±	2.58
Saline Hypo16d	41.86	±	4.41
p-value (CGRP8-37 Norm16d vs. CGRP8-37 Hypo8d)	p =	0.7855	
p-value (CGRP8-37 Norm16d vs. CGRP8-37 Hypo16d)	p =	0.0225	
p-value (CGRP8-37 Norm16d vs. Saline Hypo16d)	p <	0.0001	
p-value (CGRP8-37 Hypo8d vs. CGRP8-37 Hypo16d)	p =	0.9907	
p-value (CGRP8-37 Hypo8d vs. Saline Hypo16d)	p =	0.0225	
p-value (CGRP8-37 Hypo16d vs. Saline Hypo16d)	p =	0.7855	

Fig. 8C Vc cFos (Caud-Vc/C2 laminae I-II)

	mean number		
CGRP8-37 Norm16d	0.70	±	0.11
CGRP8-37 Hypo8d	8.01	±	1.95
CGRP8-37 Hypo16d	8.22	±	1.28
Saline Hypo16d	30.94	±	4.53
p-value (CGRP8-37 Norm16d vs. CGRP8-37 Hypo8d)	p =	0.1829	
p-value (CGRP8-37 Norm16d vs. CGRP8-37 Hypo16d)	p =	0.1485	
p-value (CGRP8-37 Norm16d vs. Saline Hypo16d)	p <	0.0001	
p-value (CGRP8-37 Hypo8d vs. CGRP8-37 Hypo16d)	p >	0.9999	
p-value (CGRP8-37 Hypo8d vs. Saline Hypo16d)	p =	0.1485	
p-value (CGRP8-37 Hypo16d vs. Saline Hypo16d)	p =	0.1829	

Fig. 8C Vc cFos (Mid-Vc laminae III-V)

	mean number		
CGRP8-37 Norm16d	0.54	±	0.19
CGRP8-37 Hypo8d	13.08	±	2.74
CGRP8-37 Hypo16d	43.03	±	7.29
Saline Hypo16d	48.28	±	9.05
p-value (CGRP8-37 Norm16d vs. CGRP8-37 Hypo8d)	p =	0.7244	
p-value (CGRP8-37 Norm16d vs. CGRP8-37 Hypo16d)	p =	0.0023	
p-value (CGRP8-37 Norm16d vs. Saline Hypo16d)	p =	0.0012	
p-value (CGRP8-37 Hypo8d vs. CGRP8-37 Hypo16d)	p =	0.2725	
p-value (CGRP8-37 Hypo8d vs. Saline Hypo16d)	p =	0.1827	
p-value (CGRP8-37 Hypo16d vs. Saline Hypo16d)	p >	0.9999	

Fig. 8C Vc cFos (Caud-Vc/C2 laminae III-V)

	mean number		
CGRP8-37 Norm16d	0.45	±	0.13
CGRP8-37 Hypo8d	7.34	±	1.42
CGRP8-37 Hypo16d	15.55	±	1.75
Saline Hypo16d	25.81	±	6.81
p-value (CGRP8-37 Norm16d vs. CGRP8-37 Hypo8d)	p =	0.6148	
p-value (CGRP8-37 Norm16d vs. CGRP8-37 Hypo16d)	p =	0.0049	
p-value (CGRP8-37 Norm16d vs. Saline Hypo16d)	p =	0.0007	
p-value (CGRP8-37 Hypo8d vs. CGRP8-37 Hypo16d)	p =	0.5185	
p-value (CGRP8-37 Hypo8d vs. Saline Hypo16d)	p =	0.1649	
p-value (CGRP8-37 Hypo16d vs. Saline Hypo16d)	p >	0.9999	

Supplementary Material Table S9

Supplementary Material Table S9 (Supplementary Material Fig. S1)

Supplementary Material Fig. S1A Body weight

	day0	day4	# p-value (vs. day0)	day8	# p-value (vs. day0)	day12	# p-value (vs. day0)	day16	# p-value (vs. day0)
Norm16d	257.60 ± 4.99	284.60 ± 5.30	p < 0.0001	306.60 ± 7.26	p < 0.0001	332.80 ± 9.15	p < 0.0001	345.80 ± 10.42	p < 0.0001
Hypo16d	235.56 ± 6.12	237.56 ± 5.99	p > 0.9999	251.33 ± 6.07	p = 0.0002	266.56 ± 6.36	p < 0.0001	261.33 ± 8.51	p < 0.0001
Hypo8d+norm8d	229.83 ± 4.36	219.00 ± 7.66	p = 0.0765	233.00 ± 10.37	p > 0.9999	277.33 ± 11.21	p < 0.0001	298.50 ± 12.01	p < 0.0001
* p-value (Norm16d vs. Hypo16d)	p = 0.1711	p = 0.0003		p < 0.0001		p < 0.0001		p < 0.0001	
* p-value (Norm16d vs. Hypo8d+norm8d)	p = 0.0834	p < 0.0001		p < 0.0001		p < 0.0001		p = 0.0008	
§ p-value (Hypo16d vs. Hypo8d+norm8d)	p > 0.9999	p = 0.2680		p = 0.2795		p = 0.9630		p = 0.0027	

Supplementary Material Fig. S1B Grip force

	day0	day4	# p-value (vs. day0)	day8	# p-value (vs. day0)	day12	# p-value (vs. day0)	day16	# p-value (vs. day0)
Norm16d	3.22 ± 0.07	3.64 ± 0.44	p = 0.9507	4.39 ± 0.37	p = 0.0058	4.76 ± 0.35	p = 0.0002	5.08 ± 0.20	p < 0.0001
Hypo16d	3.19 ± 0.14	3.98 ± 0.25	p = 0.0150	4.04 ± 0.21	p = 0.0071	4.40 ± 0.20	p < 0.0001	4.87 ± 0.23	p < 0.0001
Hypo8d+norm8d	3.13 ± 0.14	4.13 ± 0.36	p = 0.0110	3.85 ± 0.21	p = 0.1171	4.60 ± 0.15	p < 0.0001	4.28 ± 0.21	p = 0.0026
* p-value (Norm16d vs. Hypo16d)	p > 0.9999	p > 0.9999		p = 0.9786		p = 0.9195		p > 0.9999	
* p-value (Norm16d vs. Hypo8d+norm8d)	p > 0.9999	p = 0.5927		p = 0.4765		p > 0.9999		p = 0.1169	
§ p-value (Hypo16d vs. Hypo8d+norm8d)	p > 0.9999	p > 0.9999		p > 0.9999		p > 0.9999		p = 0.2439	
